# *Phyllanthus niruri* niosomes ameliorate obesity-induced hepatic steatosis in rats via modulating MALAT1/miR-206/GLP-1R signaling and hepatic lipid metabolism

**DOI:** 10.1186/s40659-026-00682-1

**Published:** 2026-04-27

**Authors:** Safaa I. Khater, Mohamed M. A. Hussein, Saydat S. Abdel-Magied, Marwa M. Lotfy, Tarek khamis, Sahar Abdelaziz, Mahmoud Mostafa, Noha Osama El-Shaer, Mahran Mohamed Abd El-Emam

**Affiliations:** 1 Department of Biochemistry and Molecular Biology, Zagazig University, Zagazig, 44511 Egypt; 2 Department of Pharmacology, Faculty of Veterinary Medicine, Zagazig University, Zagazig, 44519 Egypt; 3 Department of Pharmacognosy, Faculty of Pharmacy, Zagazig University, Zagazig, 44519 Egypt; 4 Department of Pharmaceutics, Faculty of Pharmacy, Minia University, Minya, 61519 Egypt; 5 Department of Physiology, Faculty of Medicine, Benha University, Banha, 13518 Egypt

**Keywords:** Obesity, NAFLD, *Phyllanthus niruri*, Noisome, MALAT1, GLP-1R, Lipid accumulation

## Abstract

Metabolic dysfunction associated fatty liver disease (MAFLD) is a rapidly growing global health burden characterized by hepatic lipid accumulation, insulin resistance, oxidative stress, and inflammation. Phyllanthus niruri (PHYLN) is rich in polyphenols and lignans with known antioxidant and anti-inflammatory properties; however, its therapeutic efficacy is limited by poor bioavailability. This study investigated the protective potential of PHYLN extract loaded into niosomal nanocarriers (PHYLN-NIO) against obesity-induced hepatic steatosis and explored its mechanistic similarity to glucagon-like peptide-1 receptor agonists (GLP-1RAs). Rats were divided into seven groups: control, PHYLN-NIO, HFD, HFD + Semaglutide, HFD + Semaglutide + exendin 9–39, HFD + PHYLN-NIO, and HFD + PHYLN-NIO + exendin 9–39. PHYLN-NIO markedly reduced body weight and body mass index, hepatic steatosis, and inflammation, while enhancing antioxidant status, insulin sensitivity, and lipid profiles. Mechanistically, PHYLN-NIO modulated the lncRNA-MALAT1/miR-206 axis, restored GLP-1/GLP-1R signaling, and downregulated key hepatic lipogenic regulators (LXR-α, SREBP-1c, FASN, ACC-1), while upregulating genes involved in fatty acid oxidation and metabolic homeostasis (FXR, PPAR-α, FOXA2). Notably, co-administration of exendin 9–39 reversed these effects, confirming a GLP-1R-dependent mechanism. These findings suggest that PHYLN-NIO effectively targets MALAT1/miR-206/GLP-1R signaling pathways, demonstrating its potential as a promising nanotherapeutic candidate for MAFLD management.

## Introduction

Metabolically-dysfunction-associated fatty liver disease (MAFLD) is a major worldwide health issue that is tightly correlated with oxidative stress, obesity, and insulin resistance [1]. It is also closely linked to several metabolic diseases, including type 2 diabetes mellitus (T2DM), insulin resistance (IR), and hyperlipidemia [2]. Obesity is a significant etiological factor in MAFLD, which is recognized by liver cell triglyceride accumulation exceeding 5% of weight without excessive alcohol intake [3]. Hepatic steatosis is a multifactorial ailment that results from metabolic dysfunction caused by unhealthy dietary patterns and excessive caloric intake. Alcohol consumption is another independent factor that contributes to the buildup of hepatic lipids and the advancement of the disease [4].

Semaglutide (Sema), a long-acting Glucagon-like peptide-1 receptor agonist (GLP-1-RAs), has already been validated for clinical use in the management of obesity after liraglutide [5]. It is known to benefit metabolic syndrome indicators strongly associated with MAFLD [6]. GLP-1 receptor agonists efficiently reduce fat content, body weight, and liver damage markers. Additionally, there is some evidence that these medications can promote the resolution of steatohepatitis and inhibit the development of hepatic fibrosis in NASH patients [7]. Various mechanisms are involved by GLP-1-RAs, mainly enhancing insulin secretion and lowering gastric emptying [8]. Moreover, GLP-1 RAs have anti-steatotic mechanisms through actions on the farnesoid X receptor (FXR), liver X receptor (LXR) [8], peroxisome proliferator-activated receptor gamma (PPAR-γ) expression [9], oxidative stress [9], and expression of pro-inflammatory factors [10].

Long non-coding RNAs (lncRNAs) are transcripts containing more than 200 nucleotides and are crucial for many processes, including post-transcriptional control and epigenetic changes [11]. Among lncRNAs, Metastasis Associated Lung Adenocarcinoma Transcript 1 (MALAT1) is assumed to be essential for controlling hepatic deposits of lipids [12] and liver fibrosis [13]. MALAT1 and MicroRNA-206 are linked to the initiation and progression of MAFLD and obesity due to their role in inflammation, lipid metabolism, and IR [14]. MALAT1 is frequently overexpressed in obesity and MAFLD, enhancing fat accumulation and inflammatory responses in hepatic tissues, hence worsening disease development. MiR-206, on the other hand, is a regulatory microRNA that suppresses MALAT1, reducing its lipogenic and pro-inflammatory effects. MALAT1 and miR-206 dysregulation exacerbate MAFLD and metabolic dysfunction commonly correlated with obesity by promoting chronic inflammation and hepatic fat storage [15]. Compounds present prospective targets for addressing metabolic diseases like MAFLD and obesity. By repressing MALAT1, miR-206 can reduce the expression of certain transcription factors linked to lipid accumulation and inflammatory responses. This intricate interaction between MALAT1, miR-206, and transcription factors forms a regulatory network that impacts cellular processes, especially those relevant to metabolic diseases and cancer progression, where precise control over gene expression is crucial [16–18].

Natural phytochemicals have recently been studied as potential anti-NAFLD agents [19]. *Phyllanthus niruri* (PHYLN) is a member of the Euphorbiaceae family and is abundant in tropical and subtropical climates, including parts of Sri Lanka and India. PHYLN extract is a popular Ayurvedic remedy for treating bronchitis, anemia, dermatological conditions, asthma, cough, liver, and kidney disorders [20]. PHYLN is high in flavonoids and phenolic compounds that contribute to its strong antioxidant effects [21], which could be crucial for liver protective activity [22]. PHYLN has an anti-NAFLD effect by reducing hepatic lipid peroxidation, fat deposits, and hepatic fibrosis and improving liver enzyme abnormalities [23]. Nanoscale technology has been proven to significantly enhance the therapeutic potential of medicinal plants [24]. PHYLN nanoparticles show great promise in drug delivery systems because they have antimicrobial properties that protect against *Salmonella pullorum* [25]. Furthermore, the nanoemulsion of PHYLN extract exhibits antioxidant and antibacterial properties [26].

This study assessed the potential role of PHYLN-loaded niosomes (PHYLN-NIO) in ameliorating HFD-induced hepatic steatosis in rats, emphasizing its resemblance to the action of GLP-1 receptor agonists (GLP-1RA), like Sema. The PHYLN-NIO function as GLP-1RA mimetics was validated by directly comparing their effects with Sema and further confirmed by the antagonistic reversal observed with exendin 9–39, a GLP-1R antagonist. This was verified by analyzing the mRNA expression levels of genes induced by lipogenesis, including LXR-α, FXR, SREBP-1c, FOXA2, PPAR-α, PPAR-γ, GLP-1, GLP-1r, FASN, and ACC-1, as well as their epigenetic regulation.

## Materials and methods

### Extraction of PHYLN

The aerial parts of PHYLN were purchased from Harraz for Food Industry & Natural Products, located at 1 Ahmed Maher Street, Bab Alkhalq, Cairo, Egypt. The extraction procedure was done using the method outlined by [27]. 500 g of air-dried, powdered PHYLN aerial pieces were thoroughly extracted using 80% ethanol (3 × 1.5 L). The ethanolic extract was then filtered and concentrated under vacuum, yielding 45 g of alcoholic extract (9% w/w). The extract was kept at − 20 °C for later use. The crude extract yield was calculated using the formula:

Yield (%) = (Dry weight of extract ÷ Dry weight of plant material) × 100 = (45/500) × 100 = 9%.

### Gas chromatography–mass spectrometry analysis (GC–MS)

**Fig. 1:**
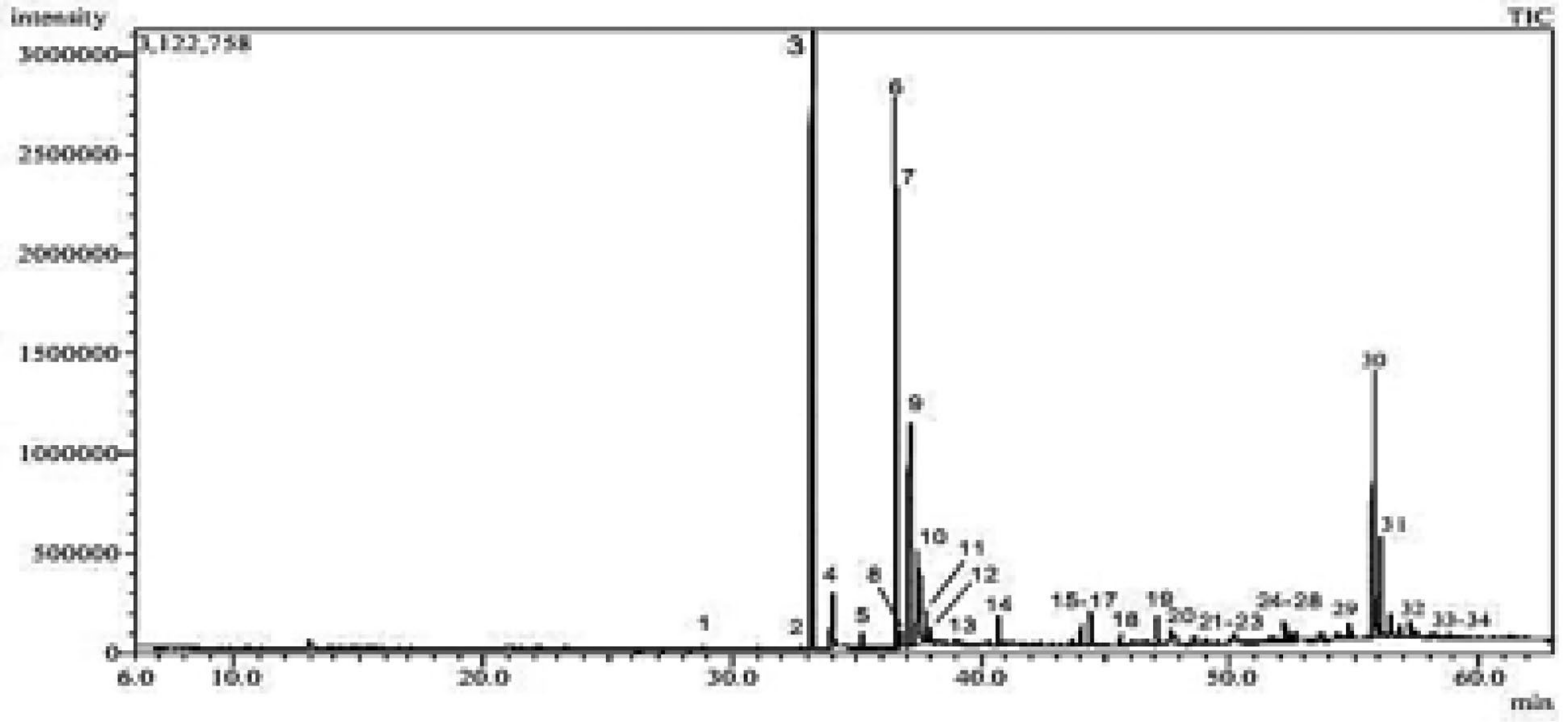
GC/MS chromatogram of PHYLN ethanolic extract. Numbers are related to Table 1

**Fig. 2:**
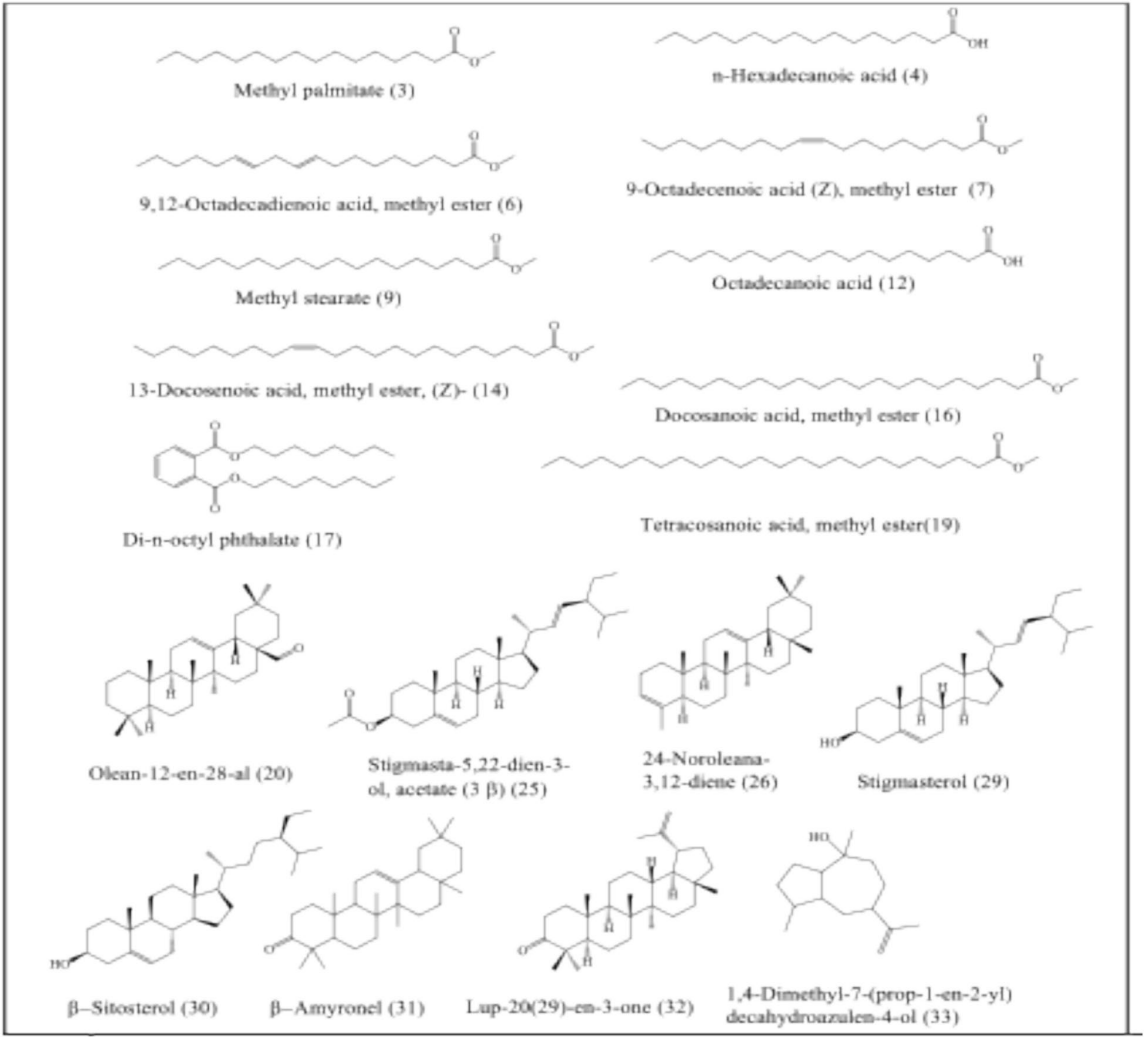
Chemical structures of the major components identified in PHYLN ethanolic extract

PHYLN GC–MS Analysis was carried out using Shimadzu GC–MS-QP2010 (Kyoto, Japan) equipped with Rtx-5MS fused bonded column (30 m × 0.25 mm i.d. × 0.25 µm film thickness) (Restek, USA) equipped with a split–splitless injector. The initial column temperature was kept at 50 °C for 3 min (isothermal) and programmed to 300 °C at a rate of 5 °C/min and kept constant at300 °C for 10 min (isothermal). Injector temperature was 280 °C. Helium carrier gas flow rate was 1.37 ml/min. All the mass spectra were recorded applying the following conditions: (equipment current) filament emission current, 60 mA; ionization voltage, 70 eV; ion source, 220 °C. Diluted samples (1% v/v) were injected with split mode (split ratio 1: 15). The constituents of PHYLN extract were based on the comparison of their mass spectra (MS) to those reported in NIST 11 Mass Spectral Library (NIST11/2011/EPA/NIH), Wiley library database 10th edition, and the available literature. The retention indices were assigned in relation to those of a homologous set of standard n-alkanes (C–C) injected under the same conditions. Table 1 summarizes the chemicals that have been identified along with their percentages (Figs. 1, 2).

**Table 1:** Chemical composition of PHYLN ethanolic extract

Peak	Compound name	Chemical formula	Retention time (min)	RI _Exp_^a^	RI _Lit_^b^	Area%
1	Tridecanoic acid, 12-methyl-, methyl ester	C_15_H_30_O_2_	28.866	1729	1615	0.11
2	9-Hexadecenoic acid, methyl ester, (Z)-	C_17_H_32_O_2_	32.751	1910	1886	0.10
3	Hexadecanoic acid, methyl ester (Methyl palmitate)	C_17_H_34_O_2_	33.205	1932	1878	**18.05**
4	n-Hexadecanoic acid	C_16_H_32_O_2_	34.044	1972	1968	**2.89**
5	Heptadecanoic acid, methyl ester	C_18_H_36_O_2_	35.186	2030	1978	0.51
6	9,12-Octadecadienoic acid, methyl ester	C_19_H_34_O_2_	36.547	2102	2093	**16.68**
7	9-Octadecenoic acid (Z)-, methyl ester	C_19_H_36_O_2_	36.660	2108	2085	**14.42**
8	11-Octadecenoic acid, methyl ester, (Z)-	C_19_H_36_O_2_	36.745	2112	2085	0.11
9	Methyl stearate	C_19_H_38_O_2_	37.116	2132	2077	**6.69**
10	cis-Vaccenic acid	C_18_H_34_O_2_	37.489	2152	2175	**8.13**
11	Linoleic acid ethyl ester	C_20_H_36_O_2_	37.770	2166	2193	0.25
12	Octadecanoic acid	C_18_H_36_O_2_	37.858	2171	2167	**1.38**
13	Nonadecanoic acid, methyl ester	C_20_H_40_O_2_	38.949	2231	2177	0.08
14	Eicosanoic acid, methyl ester	C_21_H_42_O_2_	40.709	2333	2276	**0.94**
15	13-Docosenoic acid, methyl ester, (Z)-	C_23_H_44_O_2_	43.642	2509	2483	0.15
16	Docosanoic acid, methyl ester	C_23_H_46_O_2_	44.035	2534	2475	**0.74**
17	Di-n-octyl phthalate	C_24_H_38_O_4_	44.410	2557	2832	**1.14**
18	Tricosanoic acid, methyl ester	C_24_H_48_O_2_	45.599	2634	2574	0.33
19	Tetracosanoic acid, methyl ester	C_25_H_50_O_2_	47.111	2735	2674	**0.90**
20	Olean-12-en-28-al	C_30_H_48_O	47.615	2769	2886	**0.59**
21	Pentacosanoic acid, methyl ester	C_26_H_52_O_2_	48.570	2836	2773	0.21
22	Hexadecane, 1,1-bis(dodecyloxy)-	C_40_H_82_O_2_	49.072	2872	4085	0.16
23	Hexacosanoic acid, methyl ester	C_27_H_54_O_2_	49.979	2937	2872	0.10
24	24-Noroleana-3,12-diene	C_29_H_46_	51.698	3065	2635	0.18
25	Stigmasta-5,22-dien-3-ol, acetate, (3.β.)-	C_31_H_50_O_2_	52.206	3103	2879	**0.77**
26	24-Norursa-3,12-diene	C_29_H_46_	52.330	3113	2622	**0.66**
27	Stigmasta-3,5-diene	C_29_H_48_	52.487	3125	2525	0.32
28	Octacosanoic acid, methyl ester	C_29_H_58_O_2_	52.661	3138	3071	0.34
29	Stigmasterol	C_29_H_48_O	54.808	3228	2739	**0.63**
30	β-Sitosterol	C_29_H_50_O	55.784	3353	2731	**13.21**
31	β-Amyrone	C_30_H_48_O	56.045	3370	2869	**5.49**
32	Lup-20(29)-en-3-one	C_30_H_48_O	56.844	3422	2831	**0.56**
33	1,4-Dimethyl-7-(prop-1-en-2-yl) decahydroazulen-4-ol	C_15_H_26_O	57.264	3450	1601	**1.11**
34	Stigmasta-3,5-dien-7-one	C_29_H_46_O	57.439	3461	2696	0.40

Compounds are arranged based on their elution on the Rtx-5MS GC column

^a^Retention index defined experimentally on RTX-5MS column compared to C_8_–C_28_ n-alkanes as references

^b^Reported retention index. Identification was established by matching the mass spectral (MS) data and retention index (RI) values of compounds from NIST 11 Mass Spectral Library, Wiley Registry of Mass Spectral Data 10th edition, and the literature

### Preparation and characterization of niosomal systems encapsulating PHYLN

Niosomal formulations encapsulating PHYLN have been developed using the solvent injection method, as outlined previously [28]. In this approach, a mixture of Span 60, cholesterol, polyethylene glycol 4000 (PEG_4000_), and PHYLN was dissolved in a minimal volume of anhydrous alcohol to form the organic phase, which was subsequently heated to 60–70 °C. In parallel, phosphate-buffered saline (PBS) at pH 7.4 was maintained at the same temperature and agitated at 1000 rpm within a closed system. The heated organic phase was then injected into the PBS solution through a 23G syringe, resulting in the formation of a niosomal suspension. This mixture was continuously stirred at 60–70 °C until any residual ethyl alcohol was completely evaporated. After the completion of the process, the PHYLN-loaded niosomes were stored at 4 °C for later analysis and experimentation. Blank niosomal systems were prepared following the same protocol without PHYLN. The niosomal systems were characterized by evaluating various parameters, including entrapment efficiency, drug loading efficiency, mean particle size, zeta potential, morphology using scanning electron microscopy (SEM), and in vitro drug release profiles, as described in earlier work [29]. Entrapment efficiency was determined after separating free PHYLN and assayed spectrophotometrically at 210 nm against blank niosomes.

### Animals

Fifty-six male Sprague–Dawley rats weighing 150 ± 5 g were included in this study. The rats were housed in a controlled environment with a 12-h light–dark cycle, a temperature maintained at 22 ± 3 °C, and a relative humidity of 60 ± 5%. The Institutional Animal Care and Use Committee (IACUC) of Zagazig University, Egypt (ZU-IACUC/2/F/217/2024) approved the study. The animals were assigned to seven experimental groups (n = 8). The five groups of rats were separated as follows:

1. Control group: Rats were provided with a standard diet for 18 weeks and received oral gavage with PBS once daily for the final 6 weeks. The standard pellet diet used had the following composition: 7–10% fat, 68–70% carbohydrates, 18–20% protein, 1–2% vitamins and minerals; 210 kcal/100 g/day.

2. PHYLN-NIO group: Rats were provided with a normal diet for 18 weeks and treated with PYLNI-loaded niosome (100 mg/kg body weight) via oral gavage once daily for the final 6 weeks.

3. HFD group: Rats were provided with an HFD for 12 weeks to induce NAFLD [30]. HFD consisted of 30% calories from buffalo fat (30% fat, 50–52% carbohydrates, 18–20% protein, and 1–2% vitamins and minerals; 210 kcal/100 g/day). For fatty diet preparation, fat was melted by heating it, then the chow, in powder form, was mixed with the added 30% melted animal abdominal fat until it became homogenous in a dough-like consistency. Obtained chow blocks were dried and used for feeding. These diets were prepared at the feeding department of the Faculty of Veterinary Medicine, Zagazig University.

4. HFD + Sema group: Rats were provided with an HFD and treated subcutaneously with Sema (12 μg/kg body weight) once weekly for the final 6 weeks [31].

5. HFD + Sema + exendin 9–39 group: Rats were provided with an HFD, administered Sema subcutaneously (12 μg/kg body weight) once daily for the last 6 weeks, and received intravenous injections of exendin 9–39 (50 μg/kg body weight) daily during the final 2 weeks [31].

6. HFD + PHYLN-NIO group: Rats were provided with an HFD and treated with PHYLN-NIO (100 mg/kg body weight) via oral gavage once daily for the final 6 weeks.

7. HFD + PHYLN-NIO + exendin 9–39 group: Rats were provided with an HFD and treated with PHYLN-NIO (100 mg/kg body weight) via oral gavage once daily for the last 6 weeks, along with intravenous injections of exendin 9–39 (50 μg/kg body weight) daily during the final 2 weeks.

### Sampling

After an 8-h fast, the animals were sedated with thiopental (120 mg/kg, intraperitoneally) and euthanized by decapitation. Blood samples were collected with a tube containing an anticoagulant and centrifuged at 2000 g for 10 min at 4 °C. The serum was preserved at − 80 °C for subsequent analysis. The liver was separated into three portions. One section was used for homogenization, the other one was kept in sterile cryotubes at − 80 °C for gene expression analysis, and the rest of the tissue was maintained in formaldehyde for histological and immunohistochemical examination.

### Anthropometric measurements

The body mass index (BMI) was assessed by dividing the body weight by the square of the body length, measured from the nose to the anal length in rats [32]. The formula used was BMI = body weight (g)/length^2^ (cm^2^) [33]. Liver and adipose tissue weight were measured using an electronic digital weighter.

### Biochemical analysis

Glucose levels were measured using a glucose meter (Accu-Chek Performa; Roche Diagnostics, USA). Blood insulin levels were determined by ELISA kits (RayBiotech, Georgia, USA). The HOMA-IR index was calculated as detailed in previous research [34]. Leptin levels were measured using Cusabio ELISA kits (CSB-E07433r).

Alanine aminotransferase (ALT), aspartate aminotransferase (AST), and alkaline phosphatase (ALP) were assessed using a commercial kit (BioClin®, Brazil). Quantitative detection of albumin, creatinine, and urea was performed using kits from Spinreact (Girona, Spain). Serum lipid profiles, including total cholesterol (TC), triglycerides (TAG), high-density lipoprotein cholesterol (HDL-C), and low-density lipoprotein cholesterol (LDL-C), were evaluated with a commercial KitWST (Nanjing Jiancheng, China). Very-low-density lipoprotein cholesterol (VLDL-C) levels were assessed using the formula: VLDL = TAG/5 [35]. NF-κB, IL-6, and IL-1β were quantified using ELISA assay kits from Komabiotech INC., USA, and MyBioSource, USA.

The quantification of malondialdehyde (MDA) was carried out using the technique outlined by Ohkawa et al. [36]. The total antioxidant capacity (TAC) was evaluated following Erel’s method [37]. Superoxide dismutase (SOD) activity in blood was quantified by inhibiting the formation of formazan dye at 505 nm, using the xanthine-xanthine oxidase system to produce superoxide radicals. Glutathione peroxidase (GPx) activity was assessed by quantifying the oxidation of nicotinamide adenine dinucleotide phosphate (NADPH) at 340 nm, with cumene hydroperoxide as the substrate (Randox Laboratories, Crumlin, UK).

### RNA extraction and real-time quantitative polymerase chain reaction (RT-PCR)

Total RNA was purified using TRIzol reagent (Invitrogen, CA, USA) as outlined by the manufacturer. Complementary DNA (cDNA) was assembled from 1 µg of RNA using a high-capacity cDNA reverse transcription kit (Thermo Fisher Scientific Inc., Massachusetts, USA) following the manufacturer’s instructions. For miRNA, cDNA synthesis was conducted using the miRCURY LNA RT kit (Cat. No. 339340, Qiagen, Hilden, Germany). RT-PCR analysis was carried out using the Rotor-Gene Q 2 Plex system (Qiagen, Hilden, Germany) [38]. The miRNA primers were designed using the online tool at http://www.srnaprimerdb.com, based on mature miRNA sequences available in the miRNA database https://www.mirbase.org. Primer sequences for the analyzed genes are listed in Table 1. The relative expression of miRNA and mRNA was assessed in a reaction volume of 20 µL, which included 10 µL of TOPreal SYBR Green (Cat. No. RT500S, Enzynomics, Korea) and 1 µL each of forward and reverse primers (synthesized by Sangon Biotech, Beijing, China) [37]. Gene expression was calculated relative to GAPDH and β-Actin for mRNA and U6 for miRNA using the 2–ΔΔCt method [39].

### Histopathological assessment of the liver

Liver specimens were collected and fixed in 10% neutral buffered formalin for 24 h. They were dehydrated in graded ethanol, cleared with xylene, embedded in paraffin, and sectioned into 5 μm-thick slices. The sections were dyed with hematoxylin and eosin (H&E) and analyzed microscopically for histopathological changes [40]. Images of the sections were captured using a Swift microscope equipped with a Swift digital camera. Histopathological scoring was performed using a semiquantitative scale: “0 = no alterations, 1 = mild alterations, 2 = moderate alterations, and 3 = severe alterations” [41]. The histological sections were independently evaluated by two blinded examiners to ensure unbiased analysis (Table 2).

**Table 2:** Primers used for real-time polymerase chain reaction

Genes	Forward (5′ → 3′)	Reverse (5′ → 3′)
MALAT-1	TTCACTCTAGTGCTTTATGGCTT	GGCTTCCATCCCTACATGAGA
miR-206	AACACGTGTGGAATGTAAGGAA	GTCGTATCCAGTGCAGGGT
FASN	GCAGCAGCATGATGTAGCAC	AGTTGCACACCACAAGGTCA
ACC-1	GAAAAGCGATTCCCATCCGC	CATTCCATGCAGTGGTCCCT
GLP-1	CTCAGCTCAGTCCCACAAGG	AGCTGCCTTGTACCAGCATT
GLP-1r	AGGTAGTCTTTGCCCATGCC	CCCCTAGGCAGGTTACTCCT
PPAR-α	GTCCTCTGGTTGTCCCCTTG	GTCAGTTCACAGGGAAGGCA
FXR	CCACTGACACGCCCTTTTTG	TGTTGCCGCATGGAGGATAA
PPAR-γ	TGTTGACCCAGAGCATGGTG	CACAGAGAGGTCCACAGAGC
SREBP-1c	GGAGCCATGGATTGCACATT	GCCTGTGTCTCCTGTCTCAC
FOXA2	CTGGTCGTTTGTTGTGGCTG	CGTAGTAGCTGCTCCAGTCG
LXR-α	GAGTCATCCGAGCCTACAGC	AAGAATCCCTTGCAGCCCTC
U6	CTCGCTTCGGCAGCACA	AACGCTTCACGAATTTGCGT
GAPDH	GGCACAGTCAAGGCTGAGAATG	ATGGTGGTGAAGACGCCAGTA
β-actin	AAGATCCTGACCGAGCGTGG	CAGCACTGTGTTGGCATAGAGG

### Immunohistochemical examination

Paraffin-embedded liver tissue sections from various rat groups were stained using immunohistochemistry (IHC) following the method mentioned by [42] and the manufacturer’s protocol. Anti-PPAR alpha antibody (ab59256) and anti-LXR-α antibody (ab6672) from Abcam, Cambridge, UK, were used. Tissue sections from all experimental groups were dewaxed and rehydrated before staining with the DAB chromogenic agent (Abcam; UK). Hematoxylin counterstaining was then performed. Images of the IHC-stained tissue sections were captured using a Swift microscope. For quantitative evaluation, five representative areas were chosen, including both regions with positive cell expression and areas without expression. If a tissue section exhibited areas with varying levels of stained cells, both high and low-abundance regions were included in the analysis. Individual cells were distinguished by the intense brown stain and counted manually.

### Statistical analysis

All graphs were generated using GraphPad Prism software, version 10.0.1 (San Diego, CA, USA). Data analysis was conducted using one-way ANOVA, followed by the Tukey–Kramer test. A significance level of 0.05 was established as the threshold for statistical significance.

## Results

### Characterization of PHYLN-NIO

**Fig. 3:**
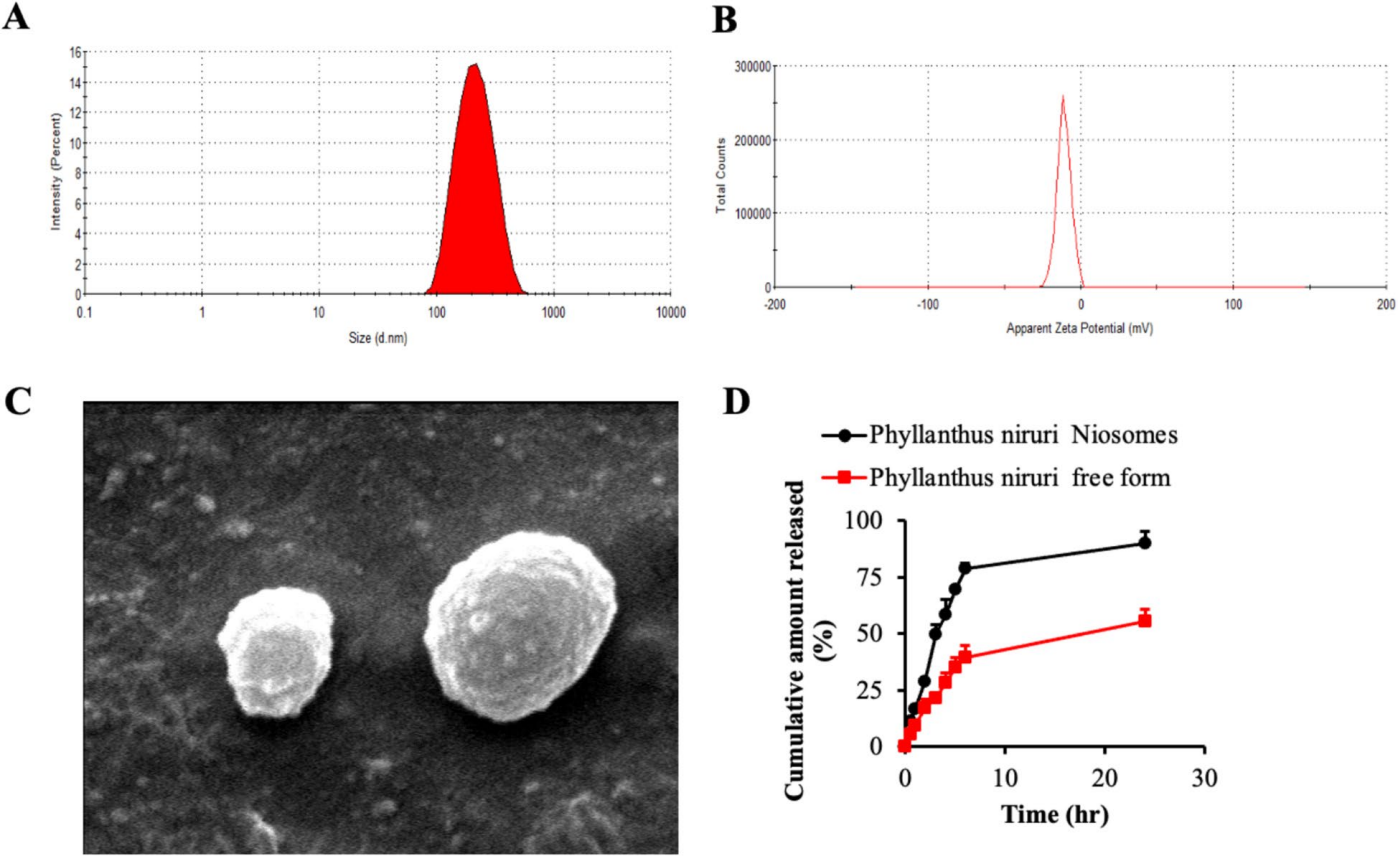
Characterization of PHYLN-NIO formulation. PHYLN-NIO was evaluated for mean particle diameter **A**, zeta potential **B**, scanning electron microscopy **C**, and in vitro drug release **D**

The ethanol injection method was employed to fabricate PHYLN-NIO, which were subsequently characterized using several analytical techniques, including particle size analysis, polydispersity index (PDI), zeta potential measurement, encapsulation efficiency, scanning electron microscopy (SEM), and in vitro drug release studies. The average particle size of the PYLNI niosomes, determined via Zetasizer Nano, was found to be 225.1 ± 19.6 nm (Fig. 3A). The polydispersity index of 0.372 indicated a relatively narrow size distribution, suggesting a uniform dispersion of the niosomal particles. The zeta potential of the niosomal systems was measured at − 11.4 ± 0.92 mV (Fig. 3B), reflecting a moderate negative surface charge, which is indicative of the stability of the formulations. SEM analysis revealed that the niosomes exhibited a predominantly spherical morphology, with the particle size closely matching that observed through dynamic light scattering measurements (Fig. 3C). The encapsulation efficiency of the PHYLN-NIO was determined to be 73.5 ± 4.2%, indicating a substantial entrapment of the active compound within the vesicles. In vitro, release studies demonstrated a sustained release profile for the PHYLN from the niosomal systems, with the free drug releasing approximately 40% of the encapsulated amount within 6 h and 55.4% after 24 h. In contrast, the PHYLN-NIO released 78% and 90.1% of the drug at 6 and 24 h, respectively, suggesting a prolonged and controlled release mechanism (Fig. 3D).

### Effects of PHYLN-NIO and Sema on BW, liver, and adipose tissue weight and BMI

A comparison of rat BW and BMI across all groups is illustrated in Table 3. No marked differences in initial BW were found between the control and PHYLN-NIO groups, but there is a considerable drop in BW and BMI in the PHYLN-NIO group compared to the control group. At baseline, no significant differences were observed between the control (150.33 ± 0.85 g) and HFD (150.10 ± 0.64 g) groups. During the first two weeks, body weight increased gradually in both groups, reaching 160.10 ± 1.2 g in controls and 161.00 ± 1.1 g in HFD rats at week 1, and 170.25 ± 1.5 g and 175.20 ± 1.6 g, respectively, at week 2. By week 3, rats fed the HFD exhibited a significant increase in body weight (210.25 ± 2.5 g) compared to the control group (180.40 ± 1.7 g, *p* < 0.05), indicating the onset of HFD-induced weight gain. By the end of the experiment, rats in the HFD group exhibited much higher BW and BMI, increasing by approximately 0.4-fold and 0.3-fold, respectively, compared to the control group. Obese rats treated with Sema or PHYLN-NIO revealed a notable reduction in BW and BMI, decreasing by about (0.4-fold and 0.3-fold), (0.2-fold, and 0.3-fold), respectively, compared to the HFD group. However, co-treatment with exendin 9–39 in the HFD + Sema and HFD + PHYLN-NIO groups caused a significant increase in BW and BMI, both rising by approximately (0.2-fold and 0.1-fold), (0.1-fold and 0.1-fold), respectively. Furthermore, rats in the HFD group exhibited much higher liver and adipose tissue weight, increasing by approximately 0.4-fold and 2.3-fold, respectively, compared to the control group. Obese rats treated with Sema or PHYLN-NIO revealed a notable reduction in liver and adipose tissue weight, decreasing by about (0.4-fold and 0.6-fold), (0.3-fold, and 0.2-fold), respectively, compared to the HFD group. However, co-treatment with exendin 9–39 in the HFD + Sema and HFD + PHYLN-NIO groups caused a significant increase in liver and adipose tissue weight. Additionally, Daily dietary intake was monitored throughout the study to determine whether changes in body weight were influenced by caloric consumption. During the 12-week experimental period, the average daily food intake of HFD-fed rats was 23.8 ± 1.2 g/day, which was slightly higher but not significantly different from the control group (22.5 ± 1.1 g/day). These results indicate that the significant weight gain observed in the HFD group was primarily due to the metabolic effects of the high-fat diet, rather than increased food consumption. Similarly, PHYLN-NIO–treated rats maintained a daily intake of approximately 22.9 ± 1.0 g/day, suggesting that the body weight–reducing effect of PHYLN-NIO occurred independently of caloric restriction.

**Table 3:** Effect of PHYLN-NIO and Sema treatment on body weight, liver, adipose weight, and BMI in HFD rats

Group	Initial body weight (g)	Final body weight (g)	Liver weight (g)	Adipose weight (g)	BMI
Control	150.33^a^ ± 0.85	355.00^d^ ± 3.68	14.20^d^ ± 0.15	28.40^e^ ± 0.29	0.75^c^ ± 0.02
PHYLN-NIO	149.32^a^ ± 1.65	311.70^e^ ± 3.33	12.47^e^ ± 0.13	31.17^de^ ± 0.33	0.64^d^ ± 0.009
HFD	150.10^a^ ± 0.64	520.00^a^ ± 4.47	20.80^a^ ± 0.18	93.60^a^ ± 0.80	1.04^a^ ± 0.01
HFD + Sema	149.60^a^ ± 2.12	315.00^e^ ± 4.18	12.60^e^ ± 0.17	31.50^d^ ± 0.42	0.68^d^ ± 0.02
HFD + Sema + exendin 9–39	150.30^a^ ± 0.96	393.40^c^ ± 6.04	15.74^c^ ± 0.24	47.21^c^ ± 0.72	0.77^c^ ± 0.01
HFD + PHYLN-NIO	150.30^a^ ± 1.64	391.60^c^ ± 4.90	15.66^c^ ± 0.20	46.99^c^ ± 0.59	0.76^c^ ± 0.03
HFD + PHYLN-NIO + exendin 9–39	151.00^a^ ± 1.70	434.40^b^ ± 5.98	17.38^b^ ± 0.24	52.13^b^ ± 0.72	0.84^b^ ± 0.02

Data are expressed as the mean ± SD (n = 8). Groups assigned different letters are deemed to differ statistically (*p* < 0.05), while groups with identical letters are not statistically different

### Impact of PHYLN-NIO and Sema on serum lipid profile levels

TC, TAG, VLDLc, HDLc, and LDLc concentrations in serum were analyzed to observe the antihyperlipidaemic effect of PHYLN-NIO in HFD-fed rats. Lipidogram is presented in Table 4. The levels of TC, TAG, VLDLc, and LDLc in the HFD group increased significantly by approximately 0.6-fold, 0.7-fold, 0.6-fold, and 1.2-fold, respectively, while HDLc levels decreased significantly by 0.3-fold, compared to the control group. Furthermore, no statistically significant differences in lipid profile levels were observed between rats treated with PHYLN-NIO alone and those in the control group. Compared to the HFD group, obese rats treated with Sema showed evident reductions in serum TC, TAG, VLDLc, and LDLc levels (approximately 0.3-fold, 0.4-fold, 0.4-fold, and 0.6-fold, respectively), along with a significant increase in HDLc levels (approximately 0.9-fold). Similarly, the HFD + PHYLN-NIO group exhibited comparable reductions in TC, TAG, VLDLc, and LDLc levels (approximately 0.3-fold, 0.4-fold, 0.4-fold, and 0.4-fold, respectively) and an increase in HDLc levels (approximately 0.3-fold). However, co-administration of exendin 9–39 with Sema and PHYLN-NIO reversed these antihyperlipidemic effects in HFD rats.

**Table 4:** Effect of PHYLN-NIO and Sema treatment on serum lipid profile in HFD rats

Group	TAG (mg/dL)	TC (mg/dL)	HDLc (mg/dL)	VLDLc (mg/dL)	LDLc (mg/dL)
Control	141.58^c^ ± 2.8	152.0^d^ ± 1.0	50.30^b^ ± 1.6	28.30^c^ ± 0.4	73.30^d^ ± 1.76
PHYLN-NIO	136^cd^ ± 0.7	146.7^d^ ± 1.37	57.30^a^ ± 0.8	27.2^cd^ ± 0.07	50.93^f^ ± 4.85
HFD	237.30^a^ ± 3.3	244.30^a^ ± 2.3	32.60^d^ ± 0.9	47.44^a^ ± 0.8	162.20^a^ ± 2.9
HFD + Sema	122.33^e^ ± 1.03	147.32^d^ ± 0.89	61.36^a^ ± 1.41	24.43^e^ ± 0.27	61.32^e^ ± 1.59
HFD + Sema + exendin 9–39	203.00^b^ ± 4.69	201.30^b^ ± 1.49	42.00^c^ ± 1.30	40.66^b^ ± 0.83	118.78^b^ ± 3.34
HFD + PHYLN-NIO	132.00^d^ ± 2.02	166.30^c^ ± 2.14	44.00^c^ ± 2.34	26.42^d^ ± 0.39	95.92^c^ ± 1.54
HFD + PHYLN-NIO + exendin 9–39	195.66^b^ ± 3.97	201.60^b^ ± 4.29	36.69^d^ ± 1.15	39.12^b^ ± 0.81	126.84^b^ ± 5.19

Data are expressed as the mean ± SD (n = 8). Groups assigned different letters are deemed to differ statistically (*p* < 0.05), while groups with identical letters are not statistically different

### The ameliorative effects of PHYLN-NIO and Sema towards changes in liver function indices in HFD rats

As illustrated in Table 5, liver enzymes ALT, AST, and ALP increased significantly by approximately 1.9-fold, 0.7-fold, and 0.6-fold each, while albumin levels decreased by about 0.1-fold in the HFD group, in contrast to the untreated group. No significant changes in liver function markers were observed between the control and PHYLN-NIO groups. Compared to the HFD group, treatment with Sema and PHYLN-NIO significantly improved liver function, reducing ALT levels by 0.5-fold and 0.5-fold and AST levels by 0.4-fold and 0.3-fold, and ALP levels by 0.3-fold and 0.3-fold, respectively, while increasing albumin levels by 0.1-fold and 0.1-fold, respectively. However, co-administration of Sema with exendin 9–39 in HFD rats reversed these improvements, resulting in a pronounced rise in ALT, AST, and ALP levels and a fall in albumin levels compared to the Sema group. Similarly, co-administration of PHYLN-NIO with exendin 9–39 in HFD rats caused an increase in ALT, AST, and ALP levels and a fall in albumin levels compared to the PHYLN-NIO group.

**Table 5:** Effect of PHYLN-NIO and Sema treatment on serum levels of AST, ALT, ALP, and albumin of HFD rats

Group	ALT (U/L)	AST (U/L)	ALP (U/L)	Albumin (gm/dl)
Control	34.70^f^ ± 0.53	57.40^cd^ ± 0.81	81.48^d^ ± 0.85	3.60^a^ ± 0.03
PHYLN-NIO	34.00^f^ ± 0.44	53.40^d^ ± 1.46	77.00^e^ ± 1.04	3.56^a^ ± 0.02
HFD	99.70^a^ ± 1.49	97.30^a^ ± 4.44	134.68^a^ ± 0.78	3.38^b^ ± 0.03
HFD + Sema	42.31^e^ ± 0.82	61.00^c^ ± 1.45	97.57^c^ ± 0.77	3.57^a^ ± 0.05
HFD + Sema + exendin 9–39	68.33^c^ ± 1.07	79.36^b^ ± 1.58	123.70^b^ ± 1.85	3.43^b^ ± 0.01
HFD + PHYLN-NIO	52.00^d^ ± 1.21	63.33^c^ ± 1.48	94.47^c^ ± 2.39	3.59^a^ ± 0.1
HFD + PHYLN-NIO + exendin 9–39	73.00^b^ ± 0.70	82.33^b^ ± 2.75	126.04^b^ ± 1.59	3.44^b^ ± 0.02

Data are expressed as the mean ± SD (n = 8). Groups assigned different letters are deemed to differ statistically (*p* < 0.05), while groups with identical letters are not statistically different

### Effect of PHYLN-NIO and Sema treatment on fasting glucose, insulin, HOMA-IR, and leptin in HFD rats

**Fig. 4:**
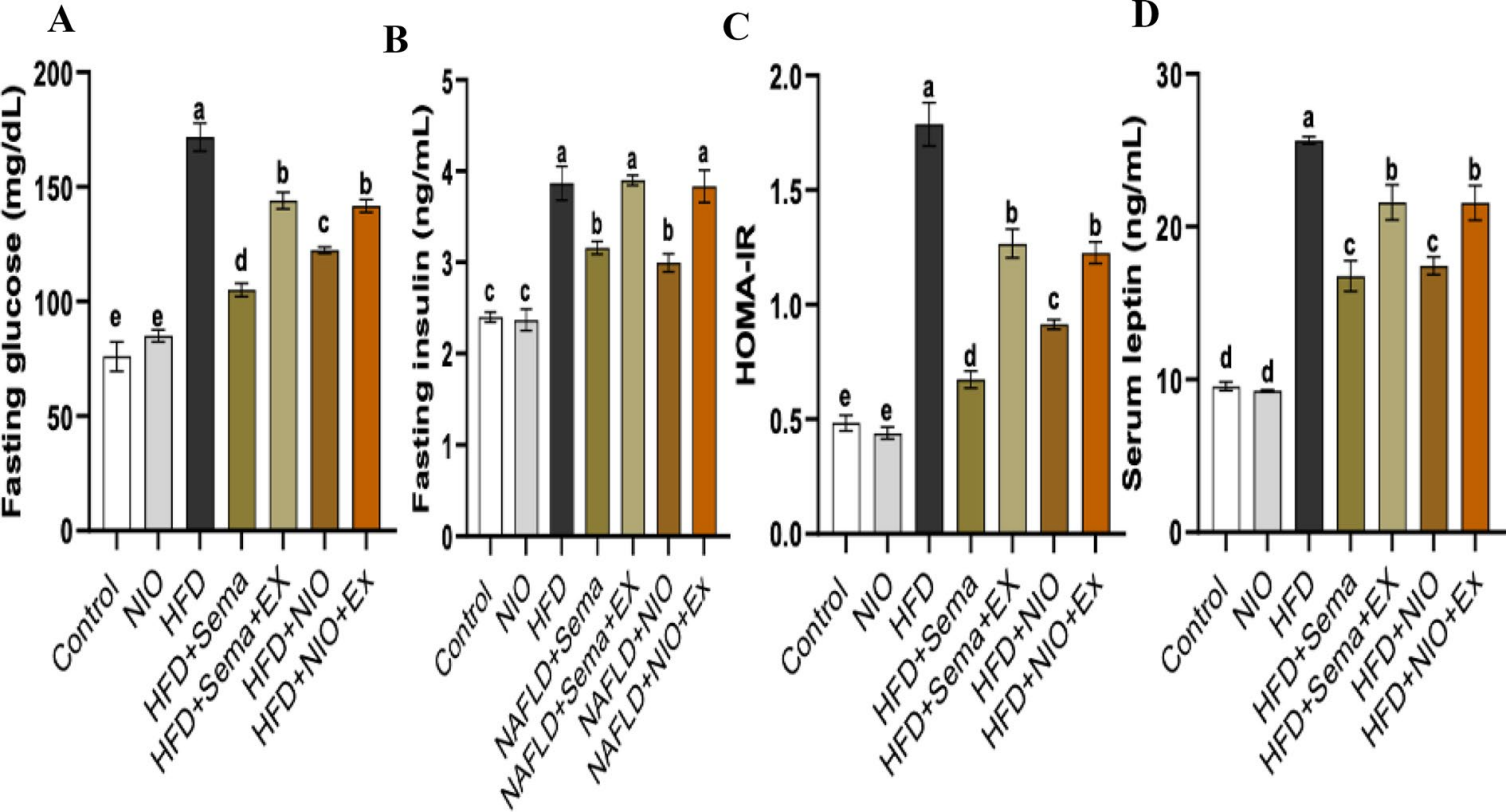
Effect of PHYLN-NIO and Sema treatment on fasting glucose **A**, insulin **B**, HOMA-IR **C**, and leptin **D** in HFD rats. Data are expressed as the mean ± SD (n = 8). Groups assigned different letters are deemed to differ statistically (*p* < 0.05), while groups with identical letters are not statistically different

Compared to the control group, HFD rats showed a notable increase in fasting glucose, insulin, HOMA-IR, and leptin levels by 0.9-fold, 0.6-fold, 2.7-fold, and 1.7-fold, respectively. No observable differences in these parameters were monitored between the PHYLN-NIO and control groups. When compared to the HFD group, administration of Sema and PHYLN-NIO significantly improved these markers, reducing fasting glucose levels by 0.4-fold and 0.3-fold, insulin levels by 0.2-fold and 0.2-fold, HOMA-IR levels by 0.6-fold and 0.5-fold, and leptin levels by 0.3-fold and 0.3-fold, respectively. However, co-administration of exendin 9–39 with either PHYLN-NIO or Sema in the HFD group negated these beneficial effects (Fig. 4A–D).

### Effect of PHYLN-NIO and Sema treatment on the oxidant/antioxidant parameters in HFD rats

As revealed in Table 6, HFD rats displayed a significant increase in hepatic MDA levels by 0.2-fold and a reduction in TAC, GPx, and SOD levels by 0.3-fold, 0.5-fold, and 0.4-fold, respectively, compared to the untreated control group. Interestingly, rats treated with PHYLN-NIO alone showed significant improvement in the antioxidant status against untreated rats. Treatment with Sema e and PHYLN-NIO led to marked reductions in hepatic MDA levels by 0.1-fold and 0.1-fold, respectively. Additionally, these treatments significantly enhanced TAC by 0.2-fold and 0.2-fold, respectively, GPx by 0.9-fold and 0.7-fold, respectively, and SOD levels by 0.6-fold and 0.5-fold, respectively, in comparison to the HFD group. However, co-administration of exendin 9–39 with either Sema or PHYLN-NIO reversed the antioxidant effects of these treatments in obese rats.

**Table 6:** Effect of PHYLN-NIO and Sema treatment on MDA, TAC, GPx, and SOD in the liver of HFD rats

Group	MDA (nmol/g)	TAC (µmol/g)	GPX (ng/g)	SOD (ng/g)
Control	103.60^e^ ± 0.81	356.40^b^ ± 2.73	161.53^b^ ± 1.18	121.95^b^ ± 0.85
PHYLN-NIO	97.60^f^ ± 1.24	371.60^a^ ± 3.38	174.21^a^ ± 0.38	128.24^a^ ± 0.67
HFD	127.00^a^ ± 1.14	249.62^e^ ± 2.96	79.93^g^ ± 0.92	71.20^g^ ± 1.13
HFD + Sema	110.00^d^ ± 1.70	314.00^c^ ± 4.38	151.58^c^ ± 2.17	112.71^c^ ± 1.00
HFD + Sema + exendin 9–39	120.00^b^ ± 0.99	292.67^d^ ± 2.28	114.94^e^ ± 1.63	95.72^e^ ± 2.50
HFD + PHYLN-NIO	115.33^c^ ± 0.66	309.00^c^ ± 1.00	134.62^d^ ± 2.32	105.80^d^ ± 1.69
N HFD + PHYLN-NIO + exendin 9–39	119.67^b^ ± 0.78	284.33^d^ ± 5.21	102.92^f^ ± 2.11	86.45^f^ ± 2.21

Data are expressed as the mean ± SD (n = 8). Groups assigned different letters are deemed to differ statistically (*p* < 0.05), while groups with identical letters are not statistically different

### The effects of PHYLN-NIO and Sema on the serum level proinflammatory cytokines induced by HFD

**Fig. 5:**
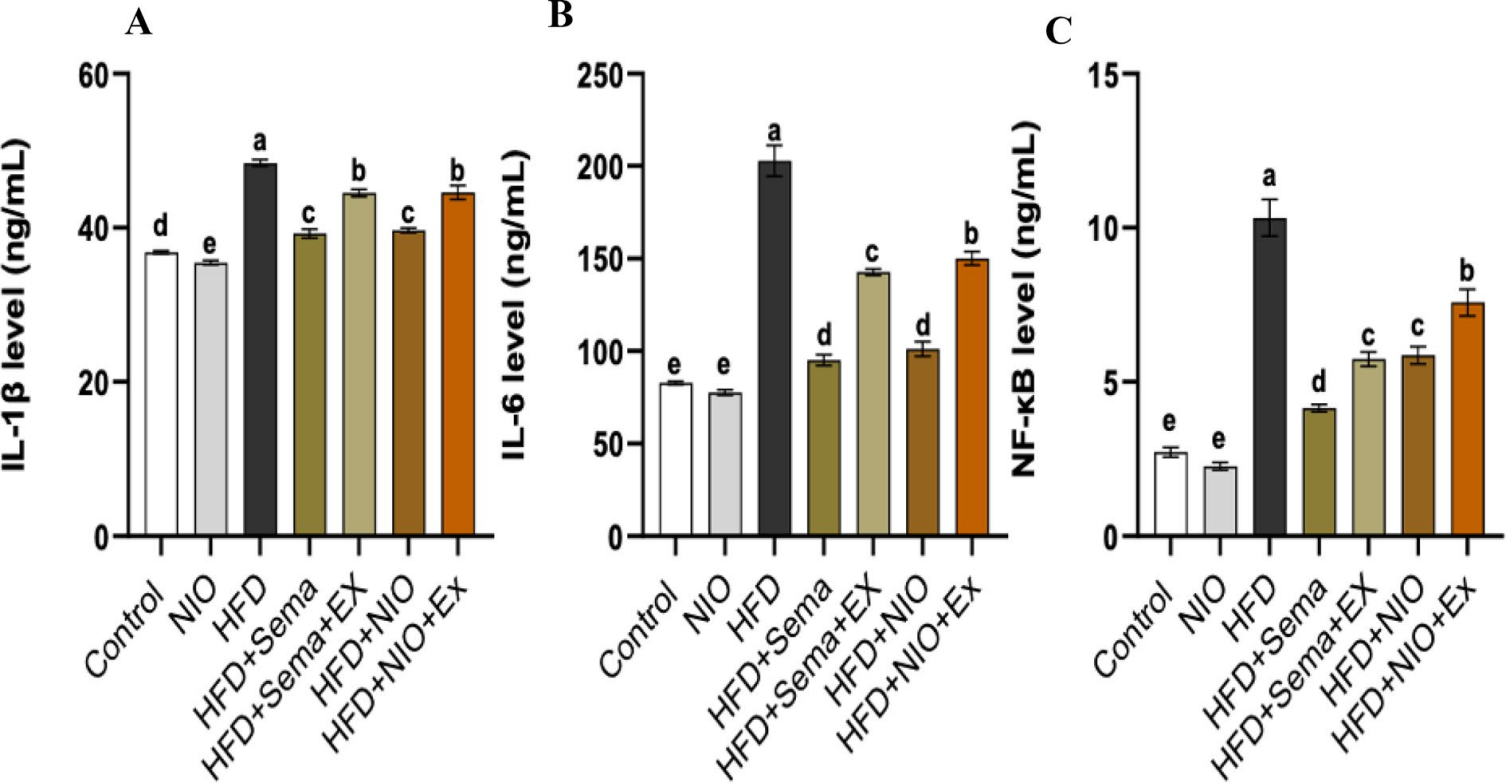
Effect of PHYLN-NIO and Sema treatment on the level of proinflammatory cytokines in the serum of HFD rats. IL-1β (A), IL-6 (B), NF-κB (C). Data are expressed as the mean ± SD (n = 8). Groups assigned different letters are deemed to differ statistically (*p* < 0.05), while groups with identical letters are not statistically different

HFD feeding caused a remarkable rise in serum levels of IL-1β, IL-6, and NF-κB by 0.3-fold, 1.4-fold, and 2.8-fold, respectively. No differences in these inflammatory markers were observed between rats treated with PHYLN-NIO alone and the control group. Treatment of HFD rats with Sema notably declined serum levels of IL-1β, IL-6, and NF-κB by 0.2-fold, 0.5-fold, and 0.6-fold, respectively, compared to the HFD group. Similarly, PHYLN-NIO treatment led to a comparable decline in these markers by 0.2-fold, 0.5-fold, and 0.4-fold, respectively. However, co-administration of exendin 9–39 with Sema or PHYLN-NIO in HFD-fed rats resulted in an additional boost in serum levels of IL-1β, IL-6, and NF-κB compared to each treatment alone (Fig. 5A–C).

### Effect of PHYLN-NIO and Sema on lncRNA-MALAT1 and miR-206 expression in hepatic tissue of HFD rats

**Fig. 6:**
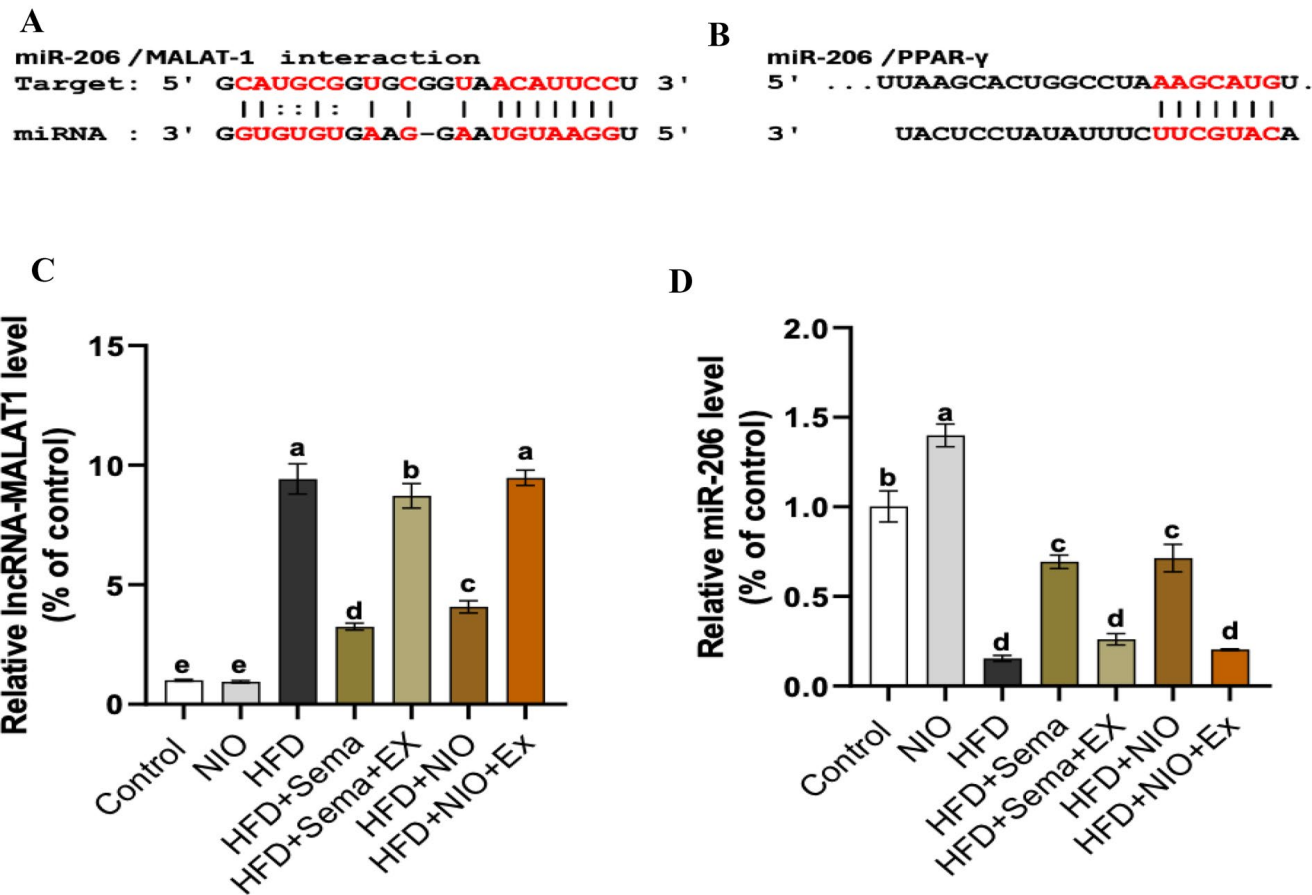
The location of the 3′ UTR region for miRNA binding to the targeted mRNA and lncRNA-*MALAT1 (A and B).* PHYLN-NIO and Sema treatment modulate expression levels of lncRNA-*MALAT1* and miR-206 (C and D) in the liver of HFD rats. Data are expressed as the mean ± SD (n = 8). Groups assigned different letters are deemed to differ statistically (*p* < 0.05), while groups with identical letters are not statistically different

The location of the 3′ UTR region for miRNA binding to the targeted mRNA and lncRNA**-**MALAT1 is indicated in Fig. 6A and B. As illustrated in Fig. 4, the HFD group demonstrated a marked rise in the mRNA expression level of lncRNA-MALAT1 by 8.4-fold, accompanied by a corresponding decrease in miR-206 mRNA expression by 0.8-fold compared to the control group. No appreciable differences were recorded in the mRNA levels of lncRNA-MALAT1 and miR-206 between rats treated with PHYLN-NIO alone and control rats. Treatment of HFD rats with Sema or PHYLN-NIO markedly reduced the mRNA expression level of lncRNA-MALAT1 by 0.6-fold and 0.5-fold and boosted the miR-206 mRNA level by 3.6-fold and 3.6-fold, respectively. However, co-administration of exendin 9–39 with Sema or PHYLN-NIO in HFD-fed rats further suppressed lncRNA-MALAT1 expression while strongly enhancing the induction of miR-206 (Fig. 6C and D).

### PHYLN-NIO and Sema balanced the mRNA expression of GLP-1 and GLP-1R in the liver of HFD rats

**Fig. 7:**
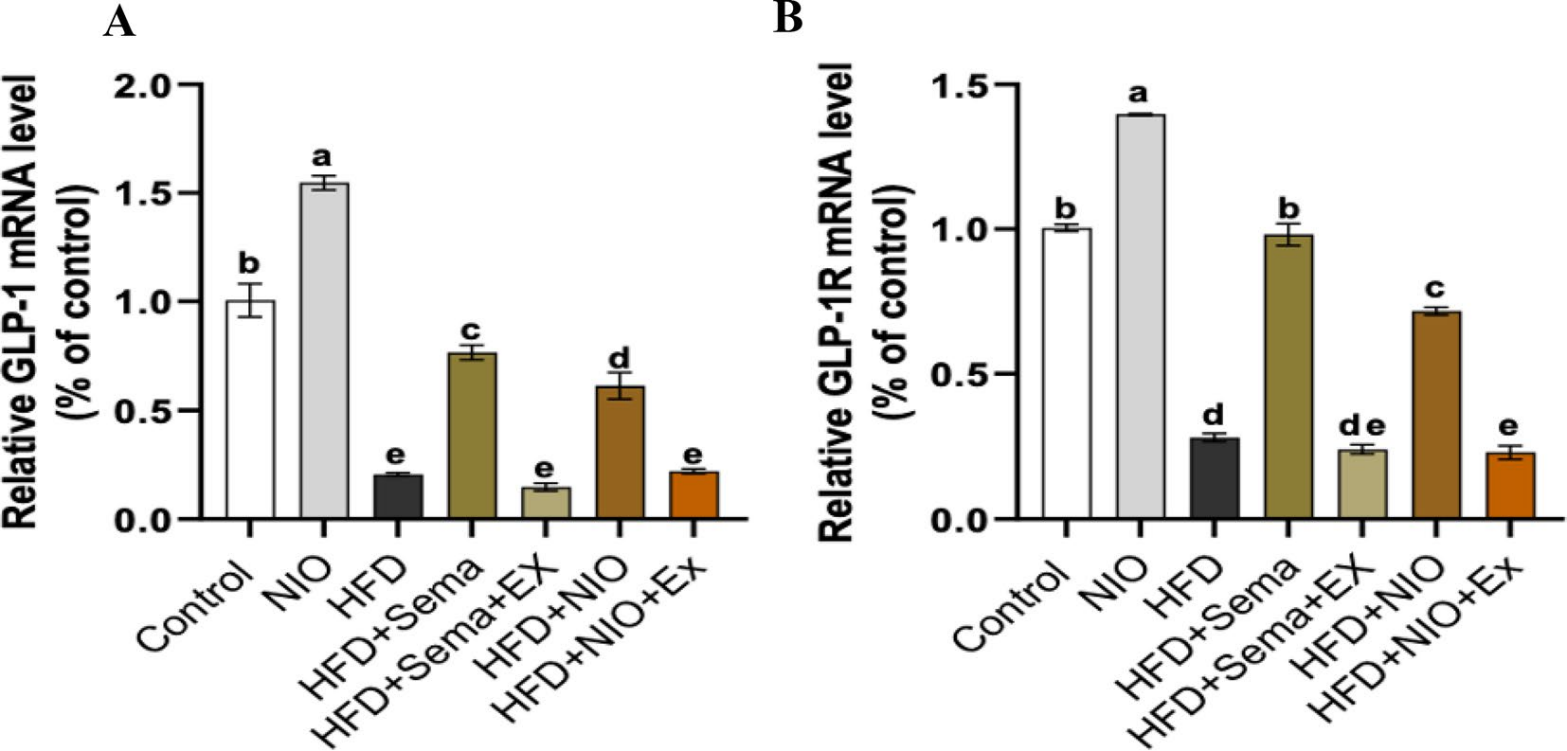
Effect of PHYLN-NIO and Sema treatment on the mRNA expression of GLP-1 and GLP-1R in the liver of HFD rats. Data are expressed as the mean ± SD (n = 8). Groups assigned different letters are deemed to differ statistically (*p* < 0.05), while groups with identical letters are not statistically different

The findings revealed that administering PHYLN-NIO alone did not influence GLP-1 and GLP-1R expression levels in rats on a normal diet. In contrast, GLP-1 and GLP-1R expression levels in HFD rats were significantly lower, by 0.8-fold and 0.7-fold, respectively, than those on a normal diet. Treatment with PHYLN-NIO markedly elevated GLP-1 and GLP-1R expression levels in HFD rats by twofold and 1.5-fold, respectively. A comparable pattern was noted in HFD-fed rats treated with Sema. However, combining exendin 9–39 with either Sema or PHYLN-NIO in HFD rats further reduced GLP-1 and GLP-1R mRNA levels compared to each treatment alone (Fig. 7A and B).

### PHYLN-NIO and Sema modulate the mRNA expression of genes involved in hepatic lipogenesis in HFD rats

**Fig. 8:**
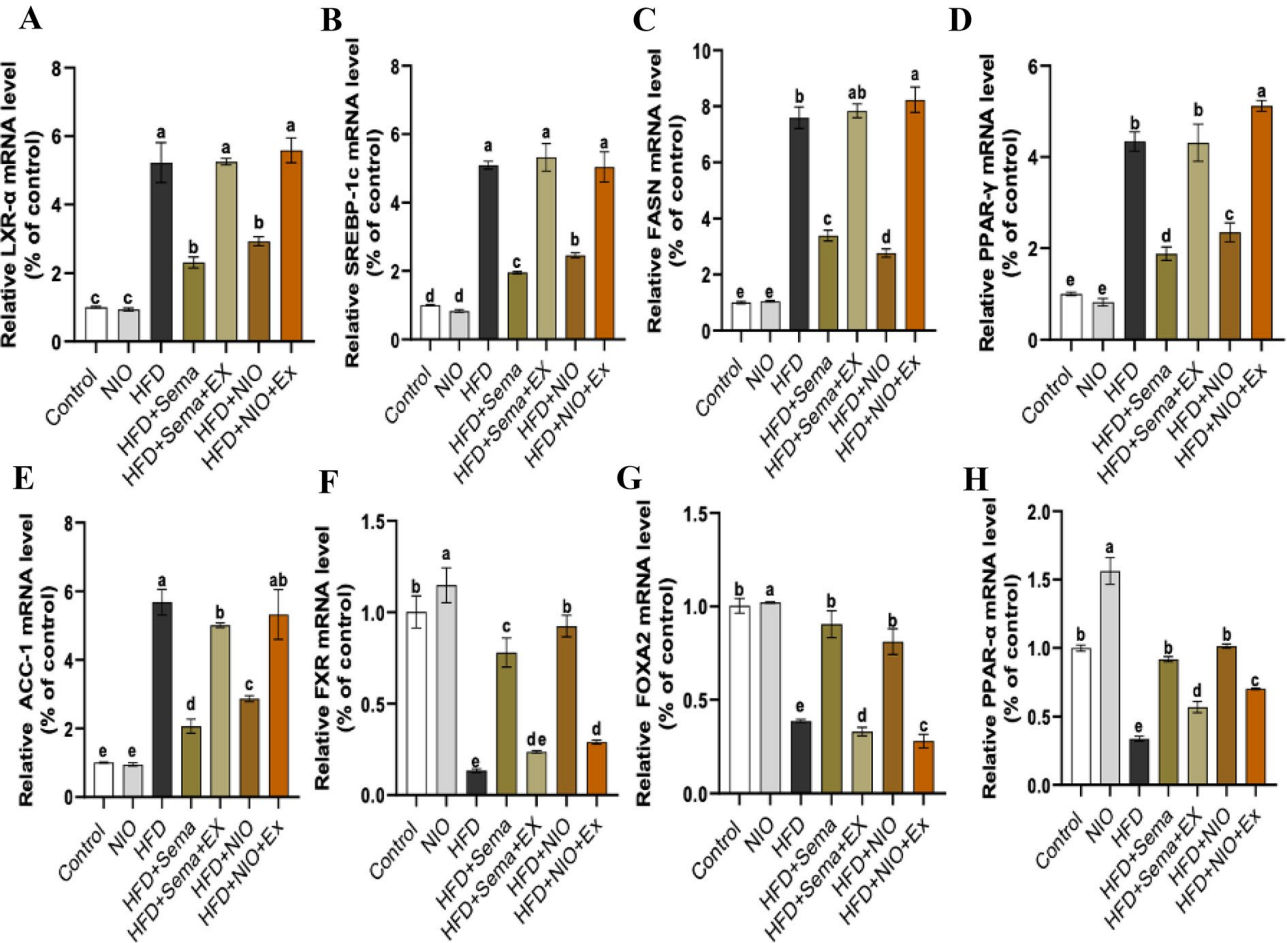
Effect of PHYLN-NIO and Sema treatment on lipogenesis-related genes in liver tissues of HFD rats. **A** LXR-α, **B** SREBP-1c, **C** PPAR-γ, **D** FASN, **E** ACC-1, **F** FXR, **G** FOXA2, and **H** PPAR-α. Data are presented as the mean ± SD (n = 7). Data are expressed as the mean ± SEM (n = 8). Groups assigned different letters are deemed to differ statistically (*p* < 0.05), while groups with identical letters are not statistically different

Figure 10 illustrates a significant rise in the mRNA levels of LXR-α, SREBP-1c, PPAR-γ, FASN, and ACC-1 (Fig. 8A–E) and a diminution in the expression of FXR, FOXA2, and PPAR-α (Fig. 8F–H), which are associated with hepatic lipogenesis in the HFD group, in contrast to the untreated group. In contrast, treatment with Sema significantly suppressed the mRNA expression of LXR-α, SREBP-1c, PPAR-γ, FASN, and ACC-1 by 0.5 fold, 0.6 fold, 0.5 fold, 0.5 fold, and 0.6 fold, respectively, while increasing the mRNA expression of FXR, FOXA2, and PPAR-α by 0.2 fold, 0.2 fold, and 0.2 fold, respectively, relative to the HFD group. Similarly, PHYLN-NIO treatment led to a comparable suppression of LXR-α, SREBP-1c, PPAR-γ, FASN, and ACC-1 by 0.4 fold, 0.5 fold, 0.4 fold, 0.6 fold, and 0.5 fold, respectively, alongside an equivalent increase in FXR, FOXA2, and PPAR-α expression by sixfold, 1.1 fold, and twofold, respectively, compared to the HFD group. However, co-administration of exendin 9–39 with either Sema or PHYLN-NIO in HFD rats resulted in increased lipogenesis compared with Sema or PHYLN-NIO alone (Fig. 8A-H).

### Immunohistochemical analysis

**Fig. 9:**
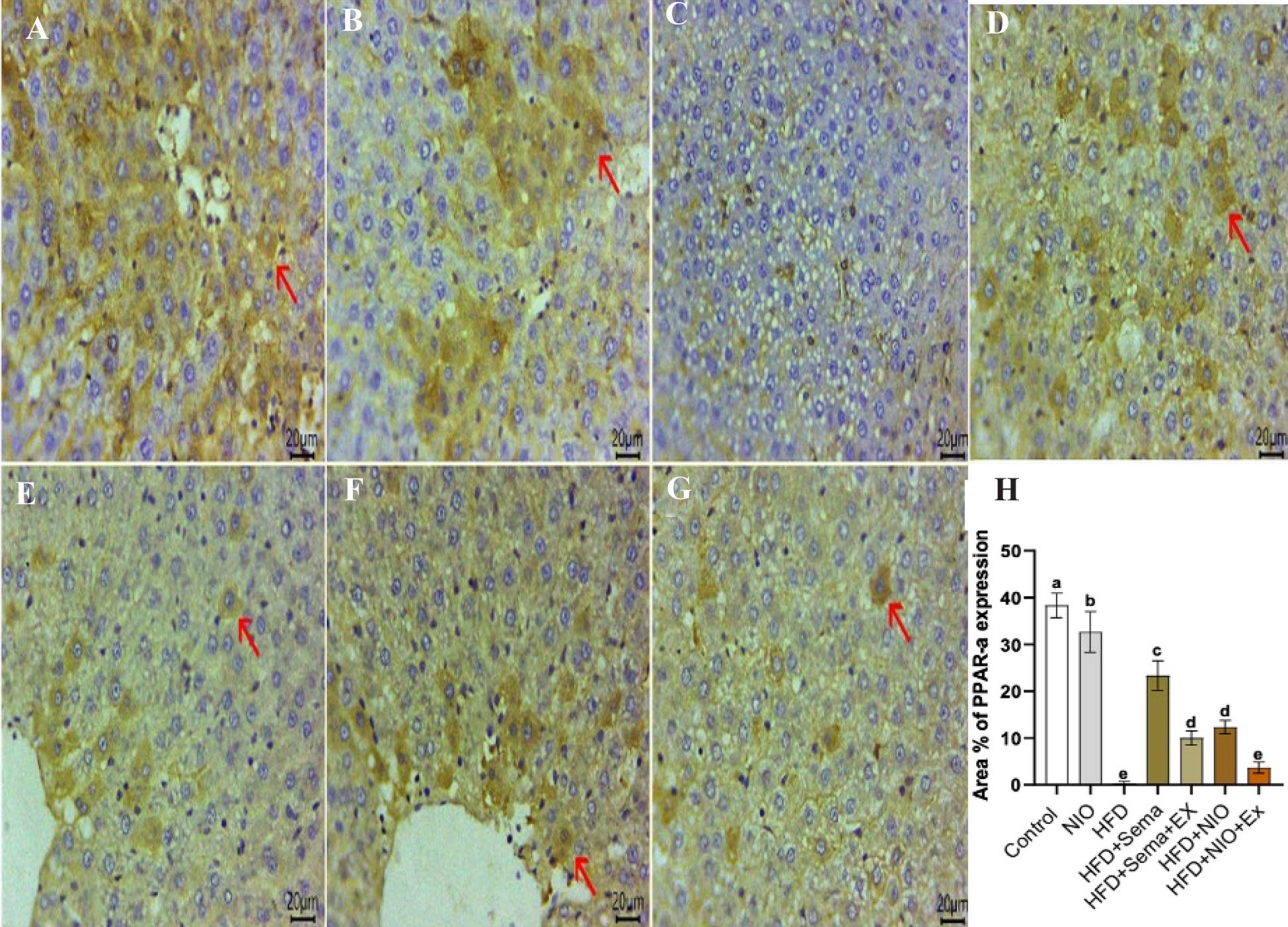
Representative photomicrographs of immunostained liver sections (Scale bar 20μm) for PPAR-α showing strong cytoplasmic expression within hepatocytes in both the control group **A** and PHYLN-NIO alone **B**. However, the HFD group displayed a complete absence of PPAR-α expression **C**. In contrast, re-established expression was observed in numerous hepatocytes in the HFD + Sema group **D**. A moderate number of PPAR- α-positive cells were detected in HFD + Sema + exendin 9–39 **E** and HFD + PHYLN-NIO group **F**, whereas HFD + PHYLN-NIO + exendin 9–39 group showed only a few immunopositive cells **G**. Graphical demonstration of the area % of PPAR-α in the different groups **H**. IHC counterstaining with Mayer’s haematoxylin. (The positive expressed cells revealed a golden-brown color). (Arrows refer to positively expressed cells)

Immunostaining of liver sections for PPAR-**α** revealed strong cytoplasmic expression within hepatocytes in both the control group (Fig. 9A) and PYLNI-loaded niosomes alone (Fig. 7B). However, the HFD group displayed a complete absence of PPAR-**α** expression (Fig. 9C). In contrast, re-established expression was observed in numerous hepatocytes in the HFD + Sema group (Fig. 9D). A moderate number of PPAR-**α**-positive cells were detected in HFD + Sema + exendin 9–39 (Fig. 7E) and HFD + PHYLN-NIO group (Fig. 9F), whereas HFD + PHYLN-NIO + exendin 9–39 group showed only a few immunopositive cells (Fig. 9G).

**Fig. 10:**
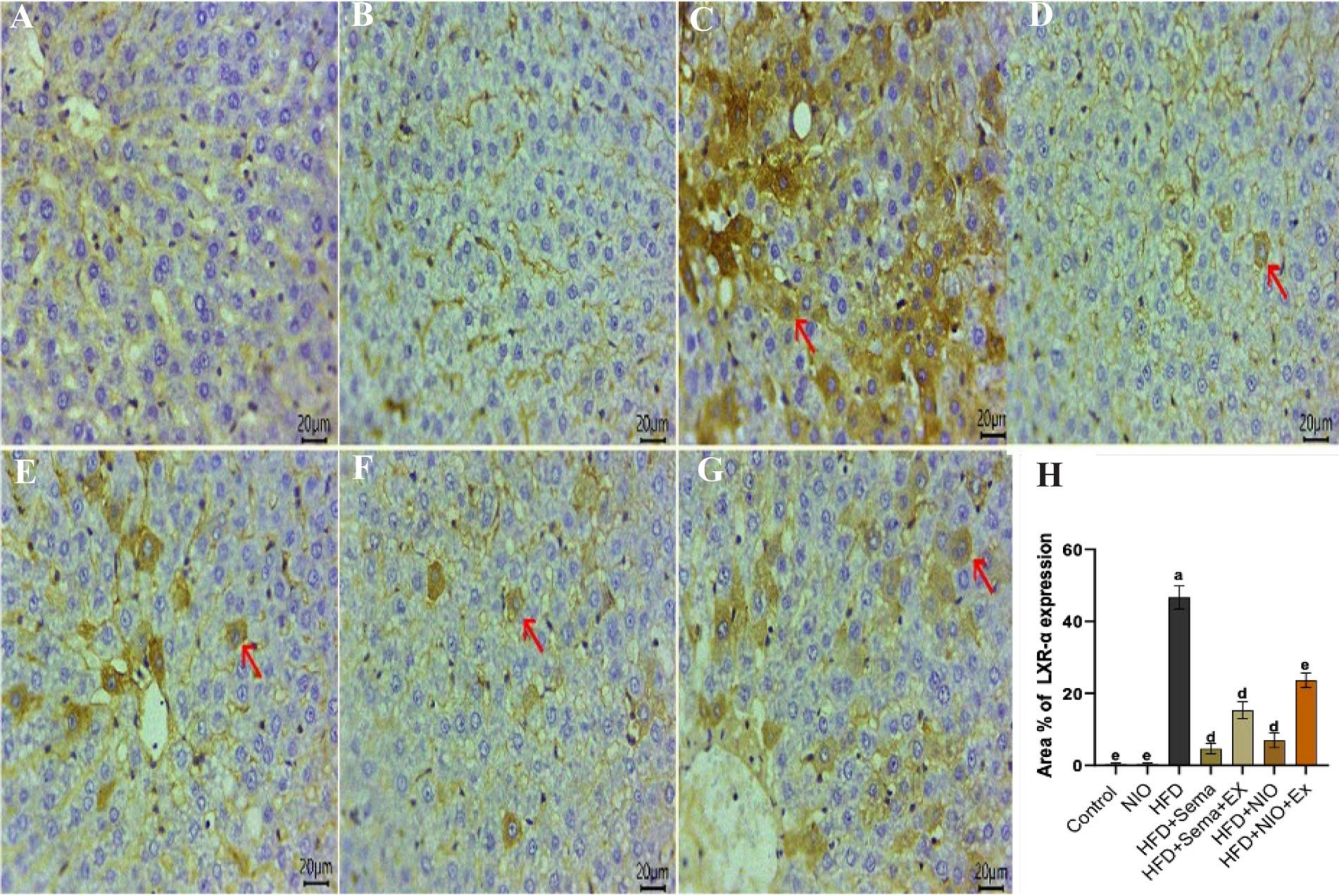
Representative photomicrographs of immunostained liver sections (Scale bar 20 μm) for LXR-α showing no expression in the control group **A** and PHYLN-NIO alone **B**. Conversely, an intense number of LXR-α-positive cells was observed in the HFD group **C**. Among the treated groups, the HFD + Sema group exhibited the lowest number of LXR-α-positive cells **D**, while moderate expression was detected in HFD + Sema + exendin 9–39 **E**. The HFD + PHYLN-NIO group showed a few positive cells **F**, and the HFD + PYLNI-loaded niosome + exendin 9–39 group had abundant cytoplasmic expression **G**, though less pronounced than in the HFD group. Graphical demonstration of the area % of LXR-α in the different groups **H**. IHC counterstaining with Mayer’s haematoxylin. (The positive expressed cells revealed a golden-brown color). (Arrows refer to positively expressed cells)

Immunohistochemical staining of liver sections for LXR-α demonstrated no expression in the control group (Fig. 10A) and PHYLN-NIO alone (Fig. 10B). Conversely, an intense number of LXR-α-positive cells was observed in the HFD group (Fig. 10C). Among the treated groups, the HFD + Sema group exhibited the lowest number of LXR-α-positive cells (Fig. 10D), while moderate expression was detected in NAFLD + Sema + exendin 9–39 (Fig. 10E). The HFD + PHYLN-NIO group showed a few positive cells (Fig. 10F), and the HFD + PHYLN-NIO + exendin 9–39 group had abundant cytoplasmic expression (Fig. 10G), though less pronounced than in the HFD group.

### Histopathological analysis of hepatic tissues

**Fig. 11:**
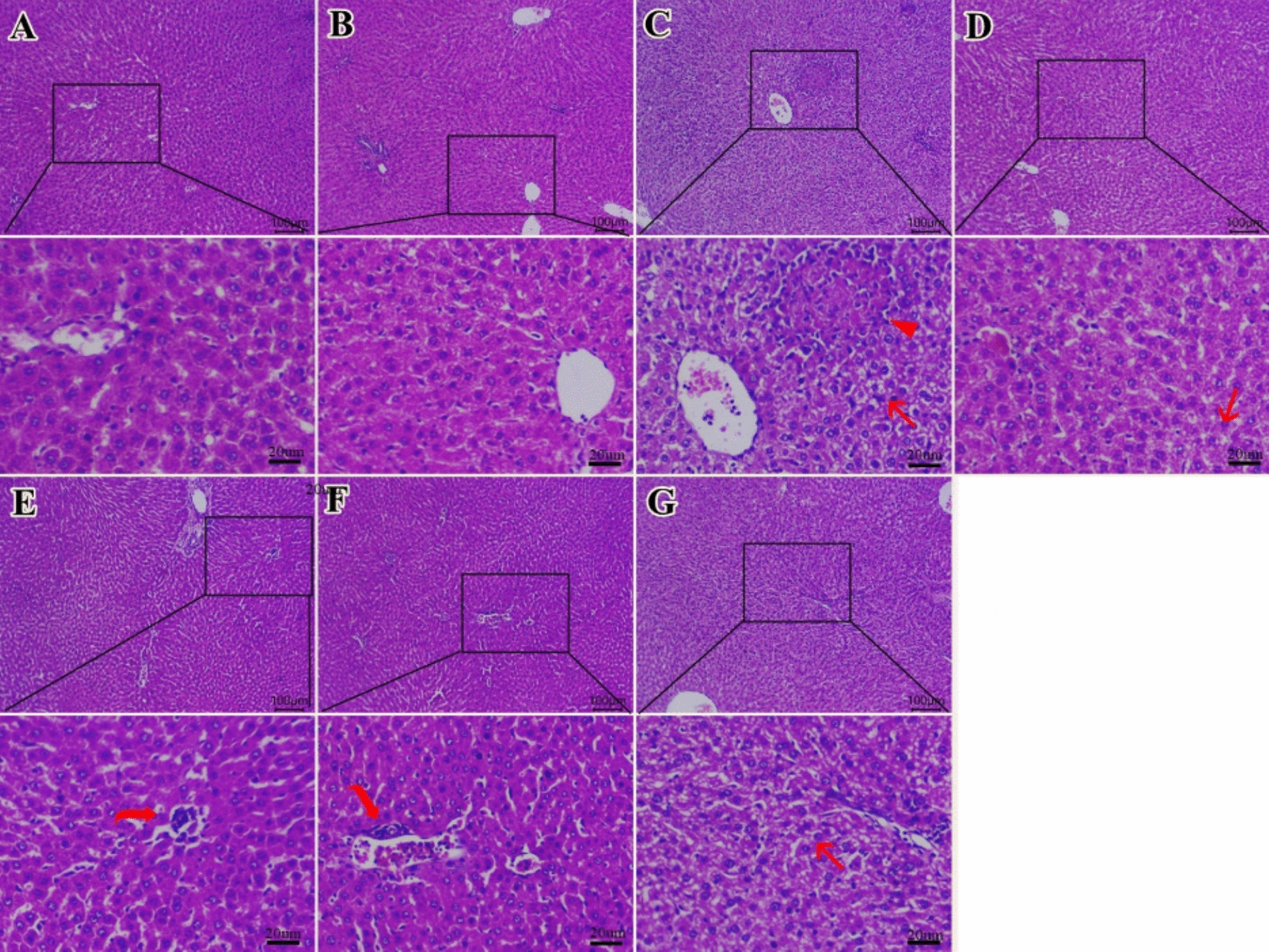
Photomicrograph of H&E-stained sections from liver sections (Scale bar 20μm). Examined sections from the liver of both the control group (**A**) and PHYLN-NIO alone (**B**) showed normal histomorphology of hepatic cells, portal areas, Kupfer cells, and central veins. However, the HFD group (**C**) revealed Diffuse areas of fatty degeneration with peripherally located nuclei (arrows). On the other hand, there are ameliorations in pathological lesions of liver tissues in all treatment groups. The HFD + Sema group (**D**) exhibited Minute fatty droplets within a few hepatocytes (arrow). HFD + Sema + exendin 9–39 (**E**) revealed a moderate degree of vacuolated hepatic cells with centrally located nuclei (arrow). The HFD + PHYLN-NIO group (**F**) exhibited a mild degree of degenerative changes within hepatic parenchyma (arrow). The worst degree of amelioration was seen in the HFD + PHYLN-NIO + exendin 9–39 group (**G**) with a moderate degree of vacuolated hepatic parenchyma (arrow) beside dilated hepatic blood vessels (arrowhead)

Examined sections from the liver of both the control group (Fig. 9A) and PHYLN-NIO alone (Fig. 9B) showed normal histomorphology of hepatic cells, portal areas, Kupfer cells, and central veins. However, the HFD group (Fig. 11C) revealed diffuse areas of fatty degeneration within hepatocytes. The fatty degenerated cells were represented by large, clear vacuoles with compressed nuclei to the periphery. On the other hand, there are ameliorations in the degenerative changes of liver tissues among different treated groups. HFD + Sema group (Fig. 11D) exhibited more pronounced ameliorations among different treated groups with minute fatty droplets within a few hepatocytes. HFD + Sema + exendin 9–39 (Fig. 11E) revealed a moderate degree of vacuolated hepatic cells with centrally located nuclei. HFD + PHYLN-NIO group (Fig. 11F) exhibited a mild degree of degenerative changes within the hepatic parenchyma. The worst degree of amelioration was seen in the HFD + PHYLN-NIO group (Fig. 11F) with a moderate degree of vacuolated hepatic cells with centrally located nuclei beside dilated hepatic blood vessels.

A scoring system for evaluating commonly observed lesions in liver tissues across different groups was illustrated in Table 7.

**Table 7:** Histopathological scoring system for evaluation of commonly observed liver lesions among different experimental groups: Liver sections were evaluated using a semi-quantitative scoring system as follows: 0 = no alteration, 1 = mild alteration, 2 = moderate alteration, and 3 = severe alteration

Lesion	Control	PHYLN-NIO	HFD	HFD + Sema	HFD + Sema + Exendin 9–39	HFD + PHYLN-NIO	HFD + PHYLN-NIO + Exendin 9–39
Hepatic steatosis	0	0	3	1	2	1	3
Focal area of necrotic hepatocytes	0	0	2	0	1	1	1
Lymphocyte infiltration	0	0	3	1	1	1	2
Inflamed portal area	0	0	2	0	2	1	1
Congested hepatic blood vessels	0	0	2	1	1	2	2

## Discussion

MAFLD is a prevalent liver disorder identified by an abnormal accumulation of liver fat without excessive alcohol consumption [2]. It comprises a range of conditions, from simple fat buildup (steatosis) to non-alcoholic steatohepatitis (NASH), which can lead to severe fibrosis and cirrhosis [43]. With the global prevalence of MAFLD, there is an urgent need for effective prevention and treatment strategies. Dietary flavonoids and phenolic compounds that exist in P. niruri exert strong anti-inflammatory and antioxidant properties, and it has been reported that using P. niruri extract has been displayed as an effective alternative therapy to ameliorate MAFLD [44]. However, the absorption pathway of P. niruri extract may be limited, affecting the distribution of its active substances. Nanotechnology is a novel and underutilized strategy to address this issue. Thus, this study proposed that the incorporation of P. niruri extract into a nanocarrier will exert a protective role in experimental rat models with obesity-induced hepatic steatosis. The efficacy of PHYLN-NIO as GLP-1RA mimetics was validated by directly comparing their effects to Sema, a widely studied GLP-1RA. Sema appears to be a promising therapeutic option for patients with NAFLD. Upon binding to its ligand, the GLP-1 receptor promotes intracellular signaling pathways that elicit various beneficial impacts. Notably, Sema has been shown to improve the parameters of metabolic syndrome, a condition strongly connected to the pathogenesis of MAFLD [45–47]. Exendin9-39, a GLP-1 receptor antagonist, was also used to investigate the GLP-1-RA-related mechanism through which PHYLN-NIO influences hepatic lipid metabolism.

In this study, HFD rats displayed a pronounced rise in BW and BMI. Similarly, [48] reported a steady rise in obesity indices and waist circumference in obese, untreated rats fed high-fat meals over six weeks. A marked increase in liver and visceral fat weight in the HFD group is a key characteristic of MAFLD [7]. Treatment with both Sema and PHYLN-NIO significantly reduced BW and BMI compared to the HFD group. Notably, PYLNI-loaded niosomes show similar efficacy to Sema, suggesting they could be a promising alternative to GLP-1RA-based therapies. However, co-treatment with exendin9-39 led to an increase in both BW and BMI, confirming the GLP-1RA-dependent mechanism of action for both treatments. PHYLN extracts effectively reduce visceral fat, liver weight, hepatic steatosis, and inflammation, thereby preventing the advancement of MAFLD [1].

It has been documented that prolonged oxidative stress is a key contributor to the initiation and advancement of MAFLD. Several studies have stated that MAFLD is accompanied by ROS overproduction or reduced antioxidant activity with subsequent oxidative stress due to an imbalance between antioxidant activity and ROS [1, 49]. Nrf2 functions as a master sensor of oxidative stress. Experimental evidence indicates that Nrf2 activation can attenuate hepatic steatosis by suppressing de novo lipogenesis and improving mitochondrial redox efficiency, whereas Nrf2 deficiency exacerbates oxidative stress, inflammation, and lipid accumulation in the liver [50]. Our findings revealed that HFD feeding disrupted the serum lipid profile, impaired liver function, and reduced antioxidant status while promoting oxidative stress and proinflammatory cytokine mediators in the HFD rat model. In detail, the marked rise in serum levels of ALP and ALT, along with elevated hepatic levels of TG, TC, and MDA, strongly indicates liver damage and oxidative stress [51]. According to the “two-hit” theory of MAFLD, increased free fatty acids are the first hit, causing lipogenesis and lipid buildup in the liver, while oxidative stress and lipid peroxidation are the second strike [52]. Consistent with our findings, accumulated fats in the liver trigger lipid peroxidation markers like MDA [53] that trigger inflammation and fibrosis [54]. Our findings showed that the HFD group showed elevated glucose levels, hyperinsulinemia, increased HOMA-IR values, marked hepatic steatosis, and inflammation. These results are consistent with other studies, demonstrating that an HFD may rapidly lead the liver to store fat and significantly raise HOMA-IR levels [55]. Elevated IR in the HFD group likely exacerbates liver oxidative stress, generating reactive oxygen species, causing hepatic lipid peroxidation, inflammatory responses, and fibrosis. This process activates hepatic stellate cells (HSC), further promoting fibrosis  [3].

In accordance with these previous observations, PHYLN-NIO and Sema effectively reduced MDA levels while increasing antioxidant markers such as TAC, GPx, and SOD. They also improved insulin sensitivity by lowering glucose, insulin levels, and IR. Furthermore, a significant reduction in TC, TG, VLDLc, and LDLc was observed. Similar effectiveness to Sema underlines PHYLN-NIO’s potential as an effective therapy for hyperlipidemia, modulating oxidative stress and ameliorating IR in HFD rats. These effects may be mediated by the phenolic chemicals found in PHYLN extracts, which decrease lipid peroxidation and increase β-oxidation and insulin signaling to mitigate oxidative stress. Similarly, previous studies have shown that polyphenols possess strong antioxidant properties, repressing lipogenesis and diminishing fatty acid synthesis in vitro [8]. Moreover, the intrinsic antioxidant properties of polyphenolics that exist in PHYLN have been ascribed to its free radical scavenging activity and thus protected against free radical-induced damage [56, 57]. Additionally, the anti-hepatic steatosis effects of PHYLN may also be attributed to ellagic acid, a potent antioxidant and a primary component of the extract. Ellagic acid is a key factor in the anti-MAFLD properties of PHYLN in vitro [23]. Furthermore, ellagic acid has also been shown to boost the expression of FFA oxidative genes in the liver, prevent TG esterification, and impair de novo lipogenesis. As a result, the buildup of hepatic lipids decreases substantially [58].

Inflammatory response, particularly in controlling the expression of cytokines implicated in inflammation, It was established that polyphenols can inhibit inflammatory responses by diminishing NF-κβ and mitogen-activated protein kinase signaling pathways [59]. Cytokines are generally recognized as key mediators of inflammation, fibrosis, and cirrhosis in NAFLD [60]. Herein, treating HFD rats with PHYLN-NIO and Sema markedly dropped serum levels of IL-1β, IL-6, and NF-κB. Similarly, Ezzat et al. [61] and [62] reported that PYLNI decreased levels of caspase-3, TNF-α, IL-1β, IL-18, and IL-10 in rats with carbon tetrachloride-induced hepatic damage. The efficient stability and higher bioavailability of PHYLN-NIO in the current study could effectively reduce the higher inflammatory response concurrent with HFD. Additionally, rats given PHYLN-NIO and Sema also revealed a notable decline in liver enzyme levels related to rats with HFD, indicating the niosomes’ strong hepatoprotective benefits. The effects were reversed by exendin 9–39 in both PHYLN-NIO and Sema groups, highlighting the GLP-1 R-dependent mechanism of action of PHYLN-NIO. The reduction in liver enzyme levels can be attributed to the polyphenolic compounds and flavonoids in PHYLN, which possess strong antioxidant properties, decreasing the adverse impacts associated with oxidative stress. These Phytochemicals like Astragalin, Gallocatechin, Ellagic acid, Gallic acid, Brevifolin carboxylic acid, Phyllnirurin, and Hypophyllanthin prevent lipid peroxidation, eliminate free radicals, and enhance the liver’s antioxidant defense system [57, 63]. Moreover, the anti-inflammatory and hepatoprotective effects of PHYLN-NIO were further supported by histopathological analysis, which revealed a mildly inflamed portal area and small foci of necrotic hepatocytes accompanied by aggregates of round cells.

Previous investigation has shown interest in the potential influence of lncRNAs in obesity [64]. LncRNAs may be crucial for controlling β-cell functionality and regulating glucose homeostasis [65]. MALAT1, a member of the lncRNA family, has numerous regulatory roles related to the expression of genes. Initially identified in several species, including humans [66]. MALAT1 acts as a potential regulator of fat deposition processes through differential gene expression analysis. This conclusion was based on its elevated expression in porcine adipose tissue, which was correlated with backfat accumulation [67]. Moreover, MiR-206 is a direct target of MALAT1 in non-small cell lung cancer, with a negative correlation observed between their expression levels [68]. Furthermore, MiR-206 is a strong inhibitor of lipid and glucose production, improving insulin signaling and suppressing hepatic lipogenesis [69]. In our investigation, lncRNA-MALAT1 mRNA expression was significantly elevated in the HFD group, while miR-206 expression was correspondingly downregulated, indicating IR and lipid accumulation in hepatic tissues. In harmony, our data showed that both PYLNI-loaded niosomes and Sema reduced the expression of lncRNA-MALAT1, promoting a rise in miR-206 mRNA levels. The downregulation of lncRNA-MALAT1 and upregulation of miR-206 indicate a potential part in controlling hepatic inflammation and lipid metabolism.

Hepatic lipogenesis in the liver is highly regulated by a network of transcription factors and enzymes. LXRs are one of the nuclear receptor (NR) class of ligand-activated transcription factors. Notably, it has been demonstrated that GLP-1 mimetics administration increases the expression of important cholesterol transporters in adipocytes via an LXR-α-mediated mechanism in 3T3-L1 adipocytes [70]. Beyond their role in cholesterol metabolism, LXRs are recognized as key regulators of hepatic fatty acid biosynthesis [71]. In addition, LXRα primarily promotes de novo lipogenesis in the liver by regulating the expression of SREBP1c, ACC1, and FAS [72]. Furthermore, PPARα and PPARγ have opposing roles in fat metabolism: PPARα facilitates fat utilization, while PPARγ activation promotes fat storage. Polyunsaturated fatty acids (PUFAs) serve as natural ligands for PPARα and PPARγ, modulating lipid metabolism and mitochondrial function. Activation of PPARα by PUFAs enhances β-oxidation of fatty acids in hepatocytes, reducing lipid accumulation and preventing steatosis. Moreover, PUFAs have been shown to improve mitochondrial biogenesis and maintain mitochondrial integrity, thereby mitigating oxidative stress and mitochondrial dysfunction commonly associated with hepatic steatosis [73].

Moreover, FXR activation lowers triglyceride levels by reducing liver fatty acid synthesis by reducing SREBP1c and LXR expression, enhancing PPARα expression, improving TG clearance, and increasing adipose tissue storage while modulating adipokine patterns [74]^.^ Adipocyte differentiation is facilitated by PPARγ-activated FXR, which promotes lipogenesis by upregulating FAS expression in adipocytes [75]. Furthermore, FoxO1 not only downregulates SREBP1c expression but also inhibits genes involved in fatty acid synthesis, like FAS and ATP citrate lyase [76]. Our study aligns with this context, as induced hyperlipidemia downregulated GLP-1 and GLP-1R expression and upregulated hepatic lipogenic genes, ultimately contributing to the progression of obesity-induced hepatic steatosis in rats.

Sema and PHYLN-NIO treatment suppressed hepatic lipogenesis by downregulating LXR-α, SREBP-1c, PPAR-γ, FASN, and ACC-1, suggesting suppression of hepatic lipogenesis while increasing FXR, FOXA2, and PPAR-α expression, which are entailed in lipid metabolism and energy homeostasis. The co-administration of exendin 9–39 with either Sema or PHYLN-NIO increased lipogenesis, like the GLP-1 and GLP-1R expression data. This implies that these lipogeneses are regulated by GLP-1 signaling through GLP-1R and that inhibiting this pathway may make hepatic lipid accumulation progressively worse. In harmony, our data showed that PHYLN-NIO treatment reduced lncRNA-MALAT1 expression, leading to increased miR-206 mRNA levels. Elevated miR-206 enhances the expression of GLP-1 and its receptor, GLP-1R. This regulation decreases hepatic lipid accumulation by influencing genes involved in lipogenesis. These effects likely contribute to PHYLN’s ability to limit pathological adipose tissue expansion, enhance insulin signaling, and reduce inflammation [1]. The PHYLN extract was found to contain several bioactive lignans, including phyllanthin, hypophyllanthin, phyltetralin, niranthin, nirtetralin, hinokinin, and isolintetralin [77], which belong to the polyphenol group and possess well-known antioxidant properties [78]. Despite the promising findings, this study has some limitations. The HFD-induced MAFLD rat model does not fully reflect the complexity of human MAFLD; therefore, clinical extrapolation should be made with caution. Although a GLP-1R–dependent mechanism was confirmed using exendin 9–39, downstream signaling pathways were not fully elucidated. In addition, the complex phytochemical composition of PHYLN extract limits attribution of the observed effects to a single compound, and possible synergistic interactions cannot be excluded. Finally, pharmacokinetic characteristics, long-term safety, and clinical validation of PHYLN-NIO were not assessed and warrant further investigation.

## Conclusion

The long-term effectiveness of nanotherapeutic approaches with antioxidant properties opens new avenues for the successful treatment of MAFLD. This study demonstrates that PHYLN-NIO effectively mitigates HFD-induced hepatic steatosis in rats. Treatment with PHYLN-NIO significantly reduced BW, BMI, oxidative stress, and inflammation. The treatment reduced the expression of lncRNA-MALAT1 while increasing miR-206 levels, enhancing GLP-1/GLP-1R signaling, and decreasing hepatic lipid accumulation. Collectively, these results emphasize the potential of PHYLN-NIO as a therapeutic approach for managing MAFLD. According to this study, PHYLN-NIO has significant clinical and nutritional significance for the treatment of MAFLD. In terms of nutrition, it encourages the use of antioxidant bioactives administered by nanotechnology to enhance redox and metabolic balance and alter gene expression related to lipid metabolism. Clinically, PHYLN-NIO shows potential as a disease-modifying therapy by reducing obesity-related parameters, oxidative stress, inflammation, and hepatic steatosis while enhancing GLP-1/GLP-1R signaling, highlighting its promise as an adjunct or alternative strategy for long-term MAFLD treatment.

## Data Availability

The data that support the findings of this study are available from the corresponding author upon reasonable request.

## References

[1] Al Zarzour RH, Ahmad M, Asmawi MZ, Kaur G, Ahmed Saeed MA, Al-Mansoub MA, et al. Phyllanthus niruri standardized extract alleviates the progression of non-alcoholic fatty liver disease and decreases atherosclerotic risk in Sprague-Dawley rats. Nutrients. 2017;9(7):766. 10.3390/NU9070766. 28718838 10.3390/nu9070766 PMC5537880

[2] Abenavoli L, Milic N, Di Renzo L, Preveden T, Medic-Stojanoska M, De Lorenzo A. Metabolic aspects of adult patients with nonalcoholic fatty liver disease. World J Gastroenterol. 2016;22(31):7006. 27610012 10.3748/wjg.v22.i31.7006 PMC4988304

[3] Videla LA, Rodrigo R, Araya J, Poniachik J. Insulin resistance and oxidative stress interdependency in non-alcoholic fatty liver disease. Trends Mol Med. 2006;12:555–8. 17049925 10.1016/j.molmed.2006.10.001

[4] Valenzuela R, Farías C, Muñoz Y, Zúñiga-Hernández J, Videla LA. Interrelationship between alcohol consumption, overnutrition, and pharmacotherapy for liver steatosis: Considerations and proposals. Mol Cell Endocrinol. 2025;611:112676. 41076005 10.1016/j.mce.2025.112676

[5] Wadden TA, Bailey TS, Billings LK, Davies M, Frias JP, Koroleva A, et al. Effect of subcutaneous semaglutide vs placebo as an adjunct to intensive behavioral therapy on body weight in adults with overweight or obesity: the STEP 3 randomized clinical trial. JAMA. 2021;325:1403–13. 33625476 10.1001/jama.2021.1831 PMC7905697

[6] Koureta E, Cholongitas E. Evolving role of semaglutide in NAFLD: in combination, weekly and oral administration. Front Pharmacol. 2024;15:1343587. 38464718 10.3389/fphar.2024.1343587 PMC10920271

[7] Nevola R, Epifani R, Imbriani S, Tortorella G, Aprea C, Galiero R, et al. GLP-1 receptor agonists in non-alcoholic fatty liver disease: current evidence and future perspectives. Int J Mol Sci. 2023;24(2):1703. 36675217 10.3390/ijms24021703 PMC9865319

[8] Lee HA, Kim HY. Therapeutic mechanisms and clinical effects of glucagon-like peptide 1 receptor agonists in nonalcoholic fatty liver disease. Int J Mol Sci. 2023;24(11):9324. 10.3390/IJMS24119324. 37298276 10.3390/ijms24119324 PMC10253495

[9] Jadhav K, Xu Y, Xu Y, Li Y, Xu J, Zhu Y, Adorini L, Lee YK, Kasumov T, Yin L, Zhang Y. Reversal of metabolic disorders by pharmacological activation of bile acid receptors TGR5 and FXR. Mol Metab 2018;9:131–40. 10.1016/j.molmet.2018.01.005 PMC5870099 29361497

[10] Niu S, Chen S, Chen X, Ren Q, Yue L, Pan X, et al. Semaglutide ameliorates metabolism and hepatic outcomes in an NAFLD mouse model. Front Endocrinol (Lausanne). 2022;13:1046130. 10.3389/FENDO.2022.1046130. 36568109 10.3389/fendo.2022.1046130 PMC9780435

[11] Robinson EK, Covarrubias S, Carpenter S. The how and why of lncRNA function: an innate immune perspective. Biochim Biophys Acta Gene Regul Mech. 2020;1863(4):194419. 10.1016/J.BBAGRM.2019.194419. 31487549 10.1016/j.bbagrm.2019.194419 PMC7185634

[12] Xiang J, Deng YY, Liu HX, Pu Y. LncRNA MALAT1 promotes PPARα/CD36-mediated hepatic lipogenesis in nonalcoholic fatty liver disease by modulating miR-206/ARNT axis. Front Bioeng Biotechnol. 2022;10:858558. 35769097 10.3389/fbioe.2022.858558 PMC9234139

[13] Sookoian S, Flichman D, Garaycoechea ME, San Martino J, Castaño GO, Pirola CJ. Metastasis-associated lung adenocarcinoma transcript 1 as a common molecular driver in the pathogenesis of nonalcoholic steatohepatitis and chronic immune-mediated liver damage. Hepatol Commun. 2018;2:654–65. 29881817 10.1002/hep4.1184 PMC5983147

[14] Aghaei SM, Hosseini SM. Inflammation-related miRNAs in obesity, CVD, and NAFLD. Cytokine. 2024;182:156724. 39106574 10.1016/j.cyto.2024.156724

[15] Qian G, Morral N. Role of non-coding RNAs on liver metabolism and NAFLD pathogenesis. Hum Mol Genet. 2022;31:R4–21. 35417923 10.1093/hmg/ddac088

[16] Farzaneh M, Najafi S, Anbiyaee O, Azizidoost S, Khoshnam SE. LncRNA MALAT1-related signaling pathways in osteosarcoma. Clin Transl Oncol. 2023;25:21–32. 35790599 10.1007/s12094-022-02876-x

[17] Ghafouri-Fard S, Azimi T, Hussen BM, Taheri M, Khoshnoud RJ, Stathopoulos C. A review on the role of non-coding RNAs in the pathogenesis of myasthenia gravis. Int j mol sci. 2021;22(23):12964. 34884767 10.3390/ijms222312964 PMC8657981

[18] Zou L, Wang X, Han X. LncRNA MALAT 1/miR-625–3p/HIF-1α axis regulates the EMT of hypoxia-induced RPE cells by activating NF-κB/Snail signaling. Exp Cell Res. 2023. 10.1016/J.YEXCR.2023.113650. 37209990 10.1016/j.yexcr.2023.113650

[19] P V V, R K S, C R M and V J V. *Research in Pharmacy*. 2011; **1**: 1–9.

[20] Dahanayake JM, Perera PK, Galappaththy P and Arawwawala M. Trends in Phytochemical Research. 2020; **3**: 101.

[21] Shajib MS, Akter S, Ahmed T, Imam MZ. Antinociceptive and neuropharmacological activities of methanol extract of Phoenix sylvestris fruit pulp. Front Pharmacol. 2015;6:212. 10.3389/FPHAR.2015.00212/FULL. 26483687 10.3389/fphar.2015.00212 PMC4591841

[22] Manjrekar AP, Jisha V, Bag PP, Adhikary B, Pai MM, Hegde A, et al. Effect of Phyllanthus niruri Linn. treatment on liver, kidney and tests in CCl4 induced hepatotoxic rats. Indian J Exp Biol. 2008;46:514–20. 18807755

[23] Lu CC, Yang SH, Hsia SM, Wu CH, Yen GC. Inhibitory effects of Phyllanthus emblica L. on hepatic steatosis and liver fibrosis in vitro. J Funct Foods. 2016;20:20–30.

[24] Sadaqa E, Utami RA, Mudhakir D. In vitro cytotoxic and genotoxic effects of Phyllanthus niruri extract loaded chitosan nanoparticles in TM4 cells and their influence on spermatogenesis. Pharmacia. 2024;71:1–14.

[25] Pharmacogn J, Hidanah S, Sabdoningrum EK, Sudjarwo SA. Phyllanthus niruri Linn) extract nanoparticle on antibacterial activity against Salmonella pullorum. Pharmacogn J. 2022;14:369–73.

[26] Pathania R, Najda A, Chawla P, Kaushik R, Khan MA. Low-energy assisted sodium alginate stabilized Phyllanthus niruri extract nanoemulsion: Characterization, in vitro antioxidant and antimicrobial application. Biotechnol Rep. 2022;33:e00711. 10.1016/j.btre.2022.e00711 PMC8850680 35198420

[27] Al-Yousef HM, Abdelaziz S, Hassan WHB, El-Sayed MA. Phytochemical and biological characterization of Tephrosia nubica boiss. Growing in Saudi Arabia. Arabian J Chem. 2020;13:9216–30.

[28] Alaaeldin E, Mostafa M, Mansour HF, Soliman GM. Spanlastics as an efficient delivery system for the enhancement of thymoquinone anticancer efficacy: Fabrication and cytotoxic studies against breast cancer cell lines. J Drug Deliv Sci Technol. 2021;65:102725.

[29] Abd El-Emam MM, Mostafa M, Farag AA, Youssef HS, El-Demerdash AS, Bayoumi H, et al. The potential effects of quercetin-loaded nanoliposomes on amoxicillin/clavulanate-induced hepatic damage: targeting the SIRT1/Nrf2/NF-κB signaling pathway and microbiota modulation. Antioxidants. 2023;12:1487. 37627483 10.3390/antiox12081487 PMC10451903

[30] Salem GA, Mohamed AAR, Khater SI, Noreldin AE, Alosaimi M, Alansari WS, et al. Enhancement of biochemical and genomic pathways through lycopene-loaded nano-liposomes: alleviating insulin resistance, hepatic steatosis, and autophagy in obese rats with non-alcoholic fatty liver disease: involvement of SMO, GLI-1, and PTCH-1 genes. Gene. 2023. 10.1016/J.GENE.2023.147670. 37516284 10.1016/j.gene.2023.147670

[31] Khamis T, Alsemeh AE, Abdullah DM. Sacubitril/valsartan (LCZ696) ameliorates hyperthyroid-induced cardiac hypertrophy in male rats through modulation of miR-377, let-7 b, autophagy, and fibrotic signaling pathways. Sci Rep. 2022;12(1):14654. 36030321 10.1038/s41598-022-18860-y PMC9420135

[32] Azemi AK, Siti-Sarah AR, Mokhtar SS, Rasool AHG. Time-restricted feeding improved vascular endothelial function in a high-fat diet-induced obesity rat model. Vet Sci. 2022. 10.3390/VETSCI9050217. 35622745 10.3390/vetsci9050217 PMC9147025

[33] Samat S, Enchang FK, Hussein FN, Ismail WIW. Four‐week consumption of Malaysian honey reduces excess weight gain and improves obesity‐related parameters in high fat diet induced obese rats. Evid Based Complement Altern Med. 2017. 10.1155/2017/1342150. 10.1155/2017/1342150 PMC5299215 28246535

[34] Wallace TM, Levy JC, Matthews DR. Use and abuse of HOMA modeling. Diabetes Care. 2004;27:1487–95. 15161807 10.2337/diacare.27.6.1487

[35] Andrade-Cetto A, Xie K, Cvejic J, Zafar M, Shajib MS, Akter S, et al. Antinociceptive and neuropharmacological activities of methanol extract of Phoenix sylvestris fruit pulp. Front Pharmacol. 2015;6:212. 26483687 10.3389/fphar.2015.00212 PMC4591841

[36] Ohkawa H, Ohishi N, Yagi K. Assay for lipid peroxides in animal tissues by thiobarbituric acid reaction. Anal Biochem. 1979;95:351–8. 36810 10.1016/0003-2697(79)90738-3

[37] Erel O. A novel automated direct measurement method for total antioxidant capacity using a new generation, more stable ABTS radical cation. Clin Biochem. 2004;37:277–85. 15003729 10.1016/j.clinbiochem.2003.11.015

[38] Abd El-Emam MM, Behairy A, Mostafa M, Khamis T, Osman NM, Alsemeh AE, et al. Chrysin-loaded PEGylated liposomes protect against alloxan-induced diabetic neuropathy in rats: the interplay between endoplasmic reticulum stress and autophagy. Biol Res. 2024;57(1):45. 38982468 10.1186/s40659-024-00521-1 PMC11232158

[39] Livak KJ, Schmittgen TD. Analysis of relative gene expression data using real-time quantitative PCR and the 2−ΔΔCT method. Methods. 2001;25:402–8. 11846609 10.1006/meth.2001.1262

[40] Bancroft JD and Layton C, *Bancrofts Theory and Practice of Histological Techniques E-Book*, 2007; 173–186.

[41] Gibson-Corley K, A O-V and undefined. *journals.sagepub.com. Veterinary pathology. journals.sagepub.com.* 2013; **50**: 1007–1015. 10.1177/0300985813485099 PMC3795863 23558974

[42] Hsu SM, Raine L, Fanger H. Use of avidin-biotin-peroxidase complex (ABC) in immunoperoxidase techniques: a comparison between ABC and unlabeled antibody (PAP) procedures. J Histochem Cytochem. 1981;29:577–80. 6166661 10.1177/29.4.6166661

[43] Petta S, Gastaldelli A, Rebelos E, Bugianesi E, Messa P, Miele L, et al. Pathophysiology of non alcoholic fatty liver disease. Int j mol sci. 2016;17(12):2082. 10.3390/ijms17122082. 27973438 10.3390/ijms17122082 PMC5187882

[44] Al Zarzour RH, Ahmad M, Asmawi MZ, Kaur G, Saeed MA, Al-Mansoub MA, et al. Phyllanthus niruri standardized extract alleviates the progression of non-alcoholic fatty liver disease and decreases atherosclerotic risk in Sprague-Dawley rats. Nutrients. 2017;9(7):766. 28718838 10.3390/nu9070766 PMC5537880

[45] Nauck MA, Quast DR. Cardiovascular safety and benefits of semaglutide in patients with type 2 diabetes: findings from SUSTAIN 6 and PIONEER 6. Front endocrinol. 2021;12:645566. 10.3389/FENDO.2021.645566. 10.3389/fendo.2021.645566 PMC8039387 33854484

[46] Gofton C, Upendran Y, Zheng MH, George J. MAFLD: how is it different from NAFLD? Clin Mol Hepatol. 2023;29:S17–31. 36443926 10.3350/cmh.2022.0367 PMC10029949

[47] Machado MV and Cortez-Pinto H. Nature Reviews Gastroenterology & Hepatology. 2022; **20**: 67–68. 10.1038/s41575-022-00717-4 36470966

[48] Arika WM, Kibiti CM, Njagi JM, Ngugi MP. Anti-obesity effects of dichloromethane leaf extract of Gnidia glauca in high fat diet-induced obese rats. Heliyon. 2019;5(11):e02800. 10.1016/J.HELIYON.2019.E02800. 31844729 10.1016/j.heliyon.2019.e02800 PMC6895710

[49] Hamad EM, Taha SH, Abou Dawood AGI, Sitohy MZ, Abdel-Hamid M. Protective effect of whey proteins against nonalcoholic fatty liver in rats. Lipid health dis. 2011;10(1):57. 10.1186/1476-511X-10-57. 10.1186/1476-511X-10-57 PMC3096574 21489294

[50] Valenzuela R, Illesca P, Echeverría F, Espinosa A, Rincón-Cervera MÁ, Ortiz M, et al. Molecular adaptations underlying the beneficial effects of hydroxytyrosol in the pathogenic alterations induced by a high-fat diet in mouse liver: PPAR-α and Nrf2 activation, and NF-κB down-regulation. Food funct. 2017;8(4):1526–37. 28386616 10.1039/c7fo00090a

[51] Xu P, Zhang X, Li Y, Yu C, Xu L, Xu G. Research on the protection effect of pioglitazone for non-alcoholic fatty liver disease (NAFLD) in rats. J Zhejiang Univ Sci B. 2006;7:627–33. 16845716 10.1631/jzus.2006.B0627 PMC1533756

[52] Lettéron P, Fromenty B, Terris B, Degott C, Pessayre D. Acute and chronic hepatic steatosis lead to in vivo lipid peroxidation in mice. J Hepatol. 1996;24:200–8. 8907574 10.1016/s0168-8278(96)80030-4

[53] Yokozawa T, Cho E, Sasaki S, A S-B and and undefined. jstage.jst.go.jp. Y SeiBiological and Pharmaceutical Bulletin. 2006.

[54] Bhattacharya D, Mukhopadhyay M, Bhattacharyya M, Karmakar P. Is autophagy associated with diabetes mellitus and its complications? A review. EXCLI J. 2018;17:709. 30190661 10.17179/excli2018-1353 PMC6123605

[55] de Bari O, Neuschwander-Tetri BA, Liu M, Portincasa P, Wang DQ. Ezetimibe: its novel effects on the prevention and the treatment of cholesterol gallstones and nonalcoholic fatty liver disease. J Lipids. 2012;2012(1):302847. 22132342 10.1155/2012/302847 PMC3216277

[56] Bhattacharjee R, Sil PC. Protein isolate from the herb, *Phyllanthus niruri* L. (Euphorbiaceae), plays hepatoprotective role against carbon tetrachloride induced liver damage via its antioxidant properties. Food Chem Toxicol. 2007;45:817–26. 17175085 10.1016/j.fct.2006.10.029

[57] Bhushan V, Bharti SK, Krishnan S, Kumar A, Kumar A. Antidiabetic effectiveness of *Phyllanthus niruri* bioactive compounds via targeting DPP-IV. Nat Prod Res. 2025. 10.1080/14786419.2024.2337108. 38590294 10.1080/14786419.2024.2337108

[58] Okla M, Kang I, Kim DM, Gourineni V, Shay N, Gu L, et al. Ellagic acid modulates lipid accumulation in primary human adipocytes and human hepatoma Huh7 cells via discrete mechanisms. J Nutr Biochem. 2015;26:82–90. 25458530 10.1016/j.jnutbio.2014.09.010

[59] Zhang H, Tsao R. Dietary polyphenols, oxidative stress and antioxidant and anti-inflammatory effects. Curr Opin Food Sci. 2016;8:33–42.

[60] Zhang T, Qin H, Wang T, Li H, Li H, Xia S, et al. Global publication trends and research hotspots of nonalcoholic fatty liver disease: a bibliometric analysis and systematic review. Springerplus. 2015;4:776. 26697286 10.1186/s40064-015-1542-1 PMC4678134

[61] Ezzat Id MI, Okba MM, Ahmed SH, El-Banna HA, Princeid A, Mohamed SO, et al. In-depth hepatoprotective mechanistic study of *Phyllanthus niruri*: in vitro and in vivo studies and its chemical characterization. PLoS ONE. 2020. 10.1371/journal.pone.0226185. 10.1371/journal.pone.0226185 PMC6961881 31940365

[62] Khamis A, Abdalla O, Hashem M, Abdelnaeim N. Comparative effects of *Phyllanthus niruri* and *Plantago major* in Carbon Tetrachloride intoxicated rats. Adv Anim Vet Sci. 2022;10:1444–50.

[63] Bagalkotkar G, Sagineedu SR, Saad MS, Stanslas J. Phytochemicals from Phyllanthus niruri Linn and their pharmacological properties: a review. J pharm pharmacol. 2006;58:1559–70. 17331318 10.1211/jpp.58.12.0001

[64] Wijesinghe SN, Nicholson T, Tsintzas K, Jones W, Jones SW. Involvements of long noncoding RNAs in obesity‐associated inflammatory diseases. Obes Rev. 2021. 10.1111/obr.13156. 33078547 10.1111/obr.13156

[65] Arnes L, Akerman I, Balderes D, J F-G and undefined. genesdev.cshlp.org. L SusselGenes & development. 2016 genesdev.cshlp.org. 10.1101/gad.273821.

[66] Hutchinson JN, Ensminger AW, Clemson CM, Lynch CR, Lawrence JB, Chess A. A screen for nuclear transcripts identifies two linked noncoding RNAs associated with SC35 splicing domains. BMC Genomics. 2007;8(1):39. 10.1186/1471-2164-8-39. 17270048 10.1186/1471-2164-8-39 PMC1800850

[67] Piórkowska K, Żukowski K, K R-M-A. of A. and undefined, sciendo.com. M TyraAnnals of Animal Science. sciendo.com. 2022, **22**, 1211–1224.

[68] Tang YC, Tian HX, Yi T, Chen HB. The critical roles of mitophagy in cerebral ischemia. Protein Cell. 2016;7:699–713. 27554669 10.1007/s13238-016-0307-0 PMC5055489

[69] Wu H, Zhang T, Pan F, Steer CJ, Li Z, Chen X, et al. MicroRNA-206 prevents hepatosteatosis and hyperglycemia by facilitating insulin signaling and impairing lipogenesis. J Hepatol. 2017;66:816–24. 28025059 10.1016/j.jhep.2016.12.016 PMC5568011

[70] Mostafa AM, Hamdy NM, El-Mesallamy HO, Abdel-Rahman SZ. Glucagon-like peptide 1 (GLP-1)-based therapy upregulates LXR-ABCA1/ABCG1 cascade in adipocytes. Biochem Biophys Res Commun. 2015;468:900–5. 26603933 10.1016/j.bbrc.2015.11.054

[71] Liang G, Yang J, Horton JD, Hammer RE, Goldstein JL, Brown MS. Diminished hepatic response to fasting/refeeding and Liver X Receptor agonists in mice with selective deficiency of Sterol Regulatory Element-binding Protein-1c. J Biol Chem. 2002;277:9520–8. 11782483 10.1074/jbc.M111421200

[72] Schultz JR, Tu H, Luk A, Repa JJ, Medina JC, Li L, et al. Role of LXRs in control of lipogenesis. Genes Dev. 2000;14:2831–8. 11090131 10.1101/gad.850400 PMC317060

[73] Echeverría F, Ortiz M, Valenzuela R, Videla LA. Long-chain polyunsaturated fatty acids regulation of PPARs, signaling: relationship to tissue development and aging. Prostaglandins Leukot Essent Fatty Acids. 2016. 10.1016/j.plefa.2016.10.001. 27926461 10.1016/j.plefa.2016.10.001

[74] Teodoro JS, Rolo AP, Palmeira CM. Hepatic FXR: key regulator of whole-body energy metabolism. Trends Endocrinol Metab. 2011;22:458–66. 21862343 10.1016/j.tem.2011.07.002

[75] Shinohara S, Fujimori K. Promotion of lipogenesis by PPARγ-activated FXR expression in adipocytes. Biochem Biophys Res Commun. 2020;527:49–55. 32446390 10.1016/j.bbrc.2020.04.075

[76] Zhang W, Patil S, Chauhan B, Guo S, Powell DR, Le J, et al. FoxO1 regulates multiple metabolic pathways in the liver. J Biol Chem. 2006;281:10105–17. 16492665 10.1074/jbc.M600272200

[77] Murugaiyah V, Chan KL. Mechanisms of antihyperuricemic effect of *Phyllanthus niruri* and its lignan constituents. J Ethnopharmacol. 2009;124:233–9. 19397979 10.1016/j.jep.2009.04.026

[78] Eklund PC, Långvik OK, Wärnå JP, Salmi TO, Willför SM, Sjöholm RE. Chemical studies on antioxidant mechanisms and free radical scavenging properties of lignans. Org Biomol Chem. 2005;3:3336–47. 16132095 10.1039/b506739a

